# Fine-grained multi-level gesture recognition based on a stretchable multichannel ultrasonic device

**DOI:** 10.1126/sciadv.aef1101

**Published:** 2026-09-23

**Authors:** Xinyi Lin, Hang Liu, Kai Lin, Jie Chen, Yuhui Huang, Jizhou Song

**Affiliations:** ^1^ Key Laboratory of Soft Machines and Smart Devices of Zhejiang Province, State Key Laboratory of Brain-Machine Intelligence, Department of Engineering Mechanics, Zhejiang University, Hangzhou 310027, China.; ^2^ Nanhu Brain-Computer Interface Institute, Zhejiang 311100, China.; ^3^ International Research Center for Information Science and Electronic Engineering, Zhejiang University, Haining 314400, China.; ^4^ State Key Laboratory of Silicon and Advanced Semiconductor Materials, School of Materials Science and Engineering, Zhejiang University, Hangzhou 310030, China.; ^5^ Department of Rehabilitation Medicine, The First Affiliated Hospital School of Medicine, Zhejiang University, Hangzhou 310003, China.

## Abstract

Discrete gesture recognition provides a direct output form for command-based human-machine interaction, while fine-grained multi-level recognition can expand command capacity by mapping subtle graded finger movements to distinct commands or different levels of the same command, thereby reducing the need for large or repetitive hand gestures. However, reliably distinguishing fine-grained multi-level gestures remains challenging. Here, we present a stretchable multichannel ultrasonic device comprising four functional sites and sixteen piezoelectric modules. Its fan-shaped substrate stretches up to 30%, enabling conformal forearm attachment and alignment with target muscle regions. Experimental characterization demonstrated sub-millimeter spatial resolution and excellent signal quality. Integrated with a one-dimensional convolutional neural network, the system achieved up to 98.75% accuracy in recognizing metacarpophalangeal joint-angle changes below 5°. The multichannel configuration yields high classification accuracy, improved class-wise recognition balance, more effective learning from multi-subject data and enhanced calibration-assisted adaptation to shifted device positions and new users, providing a reliable strategy for low-burden, fine-grained multi-level gesture command control.

## INTRODUCTION

Gesture recognition, a cornerstone of human–machine interaction, has been extensively adopted across various domains, including virtual/augmented reality (VR/AR) (*1*, *2*), smart home systems (*3*), and medical rehabilitation training (*4*, *5*). Although continuous joint-angle prediction (or hand tracking) provides detailed kinematic information, discrete gesture recognition offers a more direct output form for command-based HMI by mapping each recognized gesture state directly to a predefined command. Conventional gesture-based interfaces commonly rely on largely distinguishable hand movements, such as finger counting, hand opening and closing, or predefined symbolic gestures, to generate discrete commands. Although effective in many scenarios, these relatively large-amplitude gestures can be inconvenient in confined or socially sensitive environments and may be difficult for motor-impaired users or individuals with limited voluntary movement capacity. Therefore, fine-grained multi-level discrete-command control, in which subtle graded finger movements are mapped to multiple outputs ranging from distinct commands to different levels of the same command, represents an important interaction paradigm for human-machine interfaces.

Realizing such fine-grained multi-level command control requires sensing technologies that can reliably distinguish neighboring subtle motion states while maintaining stable performance under practical wearable conditions. Despite significant advances and demonstrated potential, various gesture recognition technologies remain constrained by inherent limitations. For instance, computer vision methods (*6*) achieve high accuracy, but rely heavily on lighting conditions, yielding poor performance under occlusion or complex backgrounds. Electromagnetic systems (*7*, *8*) are limited by spatial coverage and sensitivity to electromagnetic interference, restricting their use in mobile environments. Inertial measurement units (IMUs) based approaches (*9*, *10*) suffer from motion drift over extended periods. Although surface electromyography (sEMG) (*11*–*16*) has emerged as a prevailing solution, it remains susceptible to muscle fatigue and motion artifacts. In addition, because sEMG mainly records superficial bioelectrical activity, its limited access to deeper muscle layers may constrain its ability to distinguish subtle, closely spaced gestures. These persistent challenges highlight the need for complementary sensing strategies for fine-grained multi-level gesture recognition.

Ultrasonic sensing technology (*17*) offers a promising alternative because it is less affected by lighting conditions and visual occlusions and is capable of noninvasively capturing morphological changes in deeper muscles and tendons. Early studies (*18*–*20*) primarily used B-mode ultrasound imaging to demonstrate the feasibility of ultrasound-based gesture recognition. More recently, wearable wrist ultrasound imaging (*21*) has enabled continuous hand tracking, representing an important advance in robust wearable ultrasound-based human-machine interfaces. These studies demonstrate the strong capability of ultrasound to decode hand gestures and continuous hand motions by capturing internal muscle and tendon dynamics. Compared with B-mode ultrasound imaging, amplitude-mode (A-mode) ultrasound offers a simpler device architecture and operates through a more streamlined process involving high-frequency sound wave emission, reflection from tissue interface, and radio-frequency signal analysis, which enables targeted monitoring of structural changes in specific forearm muscles, rather than complex image reconstruction and image processing, thereby making A-mode ultrasound attractive for compact and wearable gesture-recognition applications. Huang *et al.* (*22*) showed that A-mode ultrasound achieves recognition performance comparable to that of B-mode imaging and surpasses sEMG technology. Subsequent studies (*23*–*26*) have further supported the potential of A-mode ultrasound as a next-generation solution for gesture recognition. However, current A-mode ultrasound-based devices typically employ rigid probes, which require pre-pressure to maintain tight skin contact, compromising wearability and user convenience. Flexible and stretchable ultrasound devices offer a promising technical pathway to overcome these limitations and have demonstrated broad applicability in various fields such as imaging (*27*–*29*), therapy (*30*, *31*), communication (*32*–*34*), and energy supply (*35*, *36*). Particularly, Gao *et al.* (*37*) reported a wearable single-channel A-mode ultrasound system that achieved high-precision gesture recognition with an error of only 7.9°. Despite this promising performance, the single-channel design may have limitations in two critical issues: the influence of inter-user variability and the impact of sensor positioning on recognition accuracy.

**Fig. 1.**
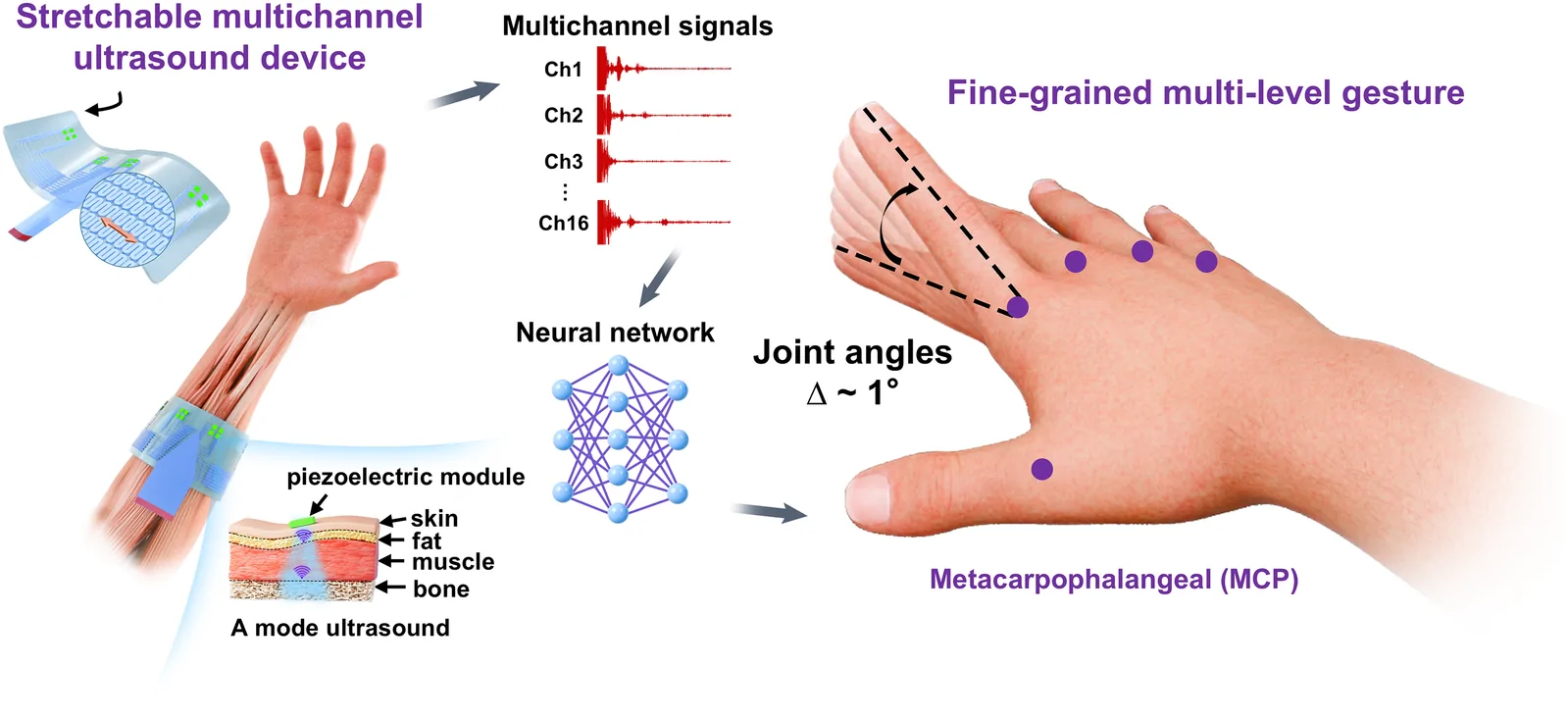
Concept of stretchable multichannel ultrasonic device for fine-grained multi-level gesture recognition. The illustrations are created by graphics software Blender and Adobe Photoshop.

Here, we report a stretchable multichannel ultrasonic device for fine-grained multi-level gesture recognition (Fig. 1). The device comprises sixteen piezoelectric modules grouped into four functional sites. Its unique fan-shaped design, combined with a stretchability of up to 30%, enables the conformal attachment to forearm while ensuring precise alignment of functional sites with target muscle regions. Experimental and numerical studies were carried out to reveal the working mechanism of device and show that with the detection depth preset to 4.92 cm, the proposed stretchable multichannel ultrasonic device can achieve a sub-millimeter resolution (461 μm) and an excellent signal-to-noise ratio (99.4% of signals exceeding 15 dB). Equipped with a one-dimensional (1D) convolutional neural network (CNN) suitable for ultrasonic signal processing, the device achieves a recognition accuracy of up to 98.75% for subtle angular changes of only 3.2° to 4.9° in the metacarpophalangeal joints. The fineness of this gesture recognition is unprecedented compared with traditional discrete gesture recognition. Further analysis indicates that the multichannel configuration transforms A-mode ultrasonic gesture recognition from local single-point sensing into spatially distributed muscle-tendon decoding, enabling high classification accuracy, improved class-wise recognition balance, and more effective learning of shared gesture-discriminative features from heterogeneous multi-subject data. In addition, the richer spatial information preserved by the multichannel model enhances the calibration-assisted adaptation to shifted sensor positions and new users. These advancements establish a foundation for more convenient and reliable fine-grained multi-level discrete-command control.

## RESULTS

### Design of stretchable multichannel ultrasonic device

**Fig. 2.**
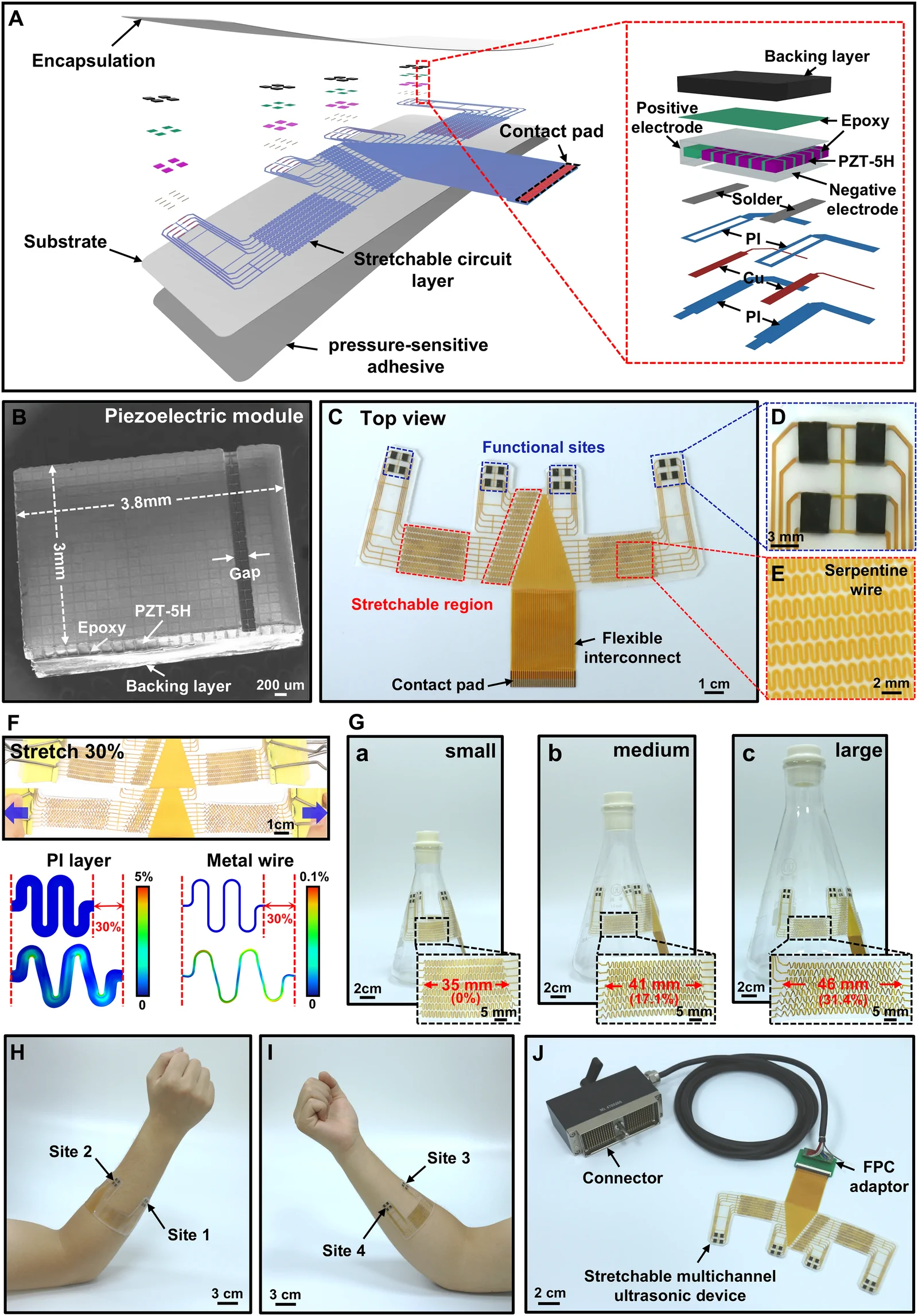
Design of stretchable multichannel ultrasonic device. (**A**) Schematic and exploded view of the stretchable multichannel ultrasonic device. (**B**) Scanning electron microscopy image depicting the piezoelectric module at an inclined angle. (**C**) Optical image of the multichannel ultrasonic device. (**D**) Optical image illustrating the design of one functional site. (**E**) Optical image of serpentine wire illustrating the design of stretchable region. (**F**) Optical image of the whole device and strain distributions in the PI (left) and Cu (right) layers of serpentine wire when uniaxially stretched to 30%. (**G**) Stretch and bend of the device demonstrating the conformal ability to conical surfaces of different curvatures. (a) Attached to a small conical flask with no stretch, (b) attached to a medium conical flask with a stretch of 17.1%, (c) attached to a large conical flask with a stretch of 31.4%. (**H** and **I**) Optical image of the device attached to the forearm. (**J**) Photograph of the stretchable multichannel ultrasonic device connected to a commercial data acquisition cable through a home-made pluggable FPC adaptor.

Figure 2A presents the exploded view of the stretchable multichannel ultrasonic device, which features sixteen piezoelectric modules grouped into four functional sites and four stretchable layers including an encapsulation layer, a circuit layer, a substrate layer, and a pressure-sensitive adhesive layer. Each piezoelectric module comprises a piezoelectric layer, an epoxy adhesive layer, and a backing layer. Figure 2B shows the scanning electron microscope (SEM) image of a piezoelectric module with dimensions of 3.8 mm by 3 mm by 1.1 mm. The piezoelectric layer adopts a high-performance anisotropic 1–3 piezoelectric composite (3.8 mm by 3 mm by 0.3 mm), which is composed of lead zirconate titanate (PZT-5H) pillars embedded in epoxy resin. Both the upper and lower surfaces of the composite are coated with nickel layers, serving as its positive and negative electrodes. The nickel layer on the side of the piezoelectric layer that connects to the backing layer is extended and flipped to the opposite side, forming a flanged electrode (i.e., positive electrode). A gap is used to separate the positive and negative electrodes (thus, the in-plane dimensions of the actual effective working area of the piezoelectric layer are 3 mm by 3 mm). The backing layer (3.8 mm by 3 mm by 0.5 mm) is bonded to positive electrode of the piezoelectric layer via an epoxy adhesive layer. It not only suppresses excessive self-oscillation of the piezoelectric layer but also expands the bandwidth, thereby enhancing signal reception performance, which is critical for ultrasonic monitoring applications. The serpentine wire, a key component of the stretchable circuit layer, features a sandwich structure of polyimide (PI: 25 μm) film-metal (Cu: 12 μm) film-polyimide (PI: 25 μm) film (fig. S1). The top PI layer is selectively patterned with openings to expose the underlying copper (Cu) electrode to connect the piezoelectric modules via low-temperature solder paste, ensuring stable electromechanical performance even when the device undergoes large deformations such as stretching and bending to adapt to complex surfaces. Figure S2 demonstrates the robust soldering effect between the piezoelectric module and the metal electrodes of the stretchable circuit. Eco-flex 00–30 is an ideal material for the substrate layer (∼50 μm) and encapsulation layer (∼1.05 mm) due to its low modulus (60 kPa), high ductility (∼900%), and the similarity of acoustic impedance to that of skin tissue (1.04 Mrayl). To ensure long-term stable adhesion, a silicone pressure-sensitive adhesive (Derma-Tac) is selected as the adhesive layer (∼13 μm), which enables effective bonding between the device and the skin, thus eliminating the need for ultrasonic coupling gel.

Figure S3 presents a schematic diagram of the fabrication process, with specific process details available in the Materials and Methods section. The fully fabricated device is shown in Fig. 2C. The device has an overall fan shape to adapt to the surface contour of the human forearm. It consists of four functional sites, three stretchable regions, flexible interconnects, and a contact pad. Each functional site comprises four piezoelectric modules, as illustrated in Fig. 2D and note S1. A 3-mm spacing is set between the piezoelectric modules, which not only enables the flexibility of the device but also ensures no cross-talk between the piezoelectric modules. The stretchable regions are composed of serpentine units with an aspect ratio of 1.64 (2.3 mm in height and 1.4 mm in width), as shown in Fig. 2E. To ensure the positional stability of devices and the reliability of signal measurements, we implemented asymmetric serpentine interconnect structures only in three designated regions (note S2). Figure 2F shows that this design allows the device to achieve a 30% stretch without fracture. Finite element analysis (FEA) indicates that the maximum principal strains of the PI layer (4.18%) and the metal layer (0.088%) are far below their respective damage limits [9.8% and 1% (*38*)], respectively, confirming the safety of the device under large deformations.

Ultrasonic devices are typically designed for attachment to body surface projections overlying different muscles within the same cross-section of the forearm (*25*, *39*–*41*). As demonstrated in Fig. 2G, the proposed stretchable multichannel ultrasonic device exhibits excellent conformability to flasks of varying curvatures due to its excellent stretchability. Deviations of functional sites from target positions when ignoring the stretchability of serpentine wires are shown in fig. S4. This unique property indicates the device’s adaptability to individuals with different forearm sizes. Furthermore, as shown in Fig. 2, H and I, the device can be easily and accurately positioned onto target muscle sites on the forearm. Figure S5 presents the top and bottom views of the contact pad. The top side exposes the metal electrodes for electrical connection to the adapter board, while the bottom incorporates a 300-μm-thick reinforcement layer that facilitates repeated plugging and unplugging and ensures a reliable connection. Figure 2J shows a schematic diagram of the connection between the stretchable multichannel ultrasonic device and a commercial ultrasonic signal acquisition cable via a custom-designed circuit adapter board. The cable is then connected to the Verasonics ultrasonic acquisition system (fig. S6), where a customized program enables real-time acquisition of ultrasonic signals.

### Characterization of the stretchable multichannel ultrasonic device

Previous studies have reported that transducers with a resonance frequency of 5 MHz exhibit superior signal transmission and reception capabilities (*42*, *43*). In this study, 5 MHz was selected as the frequency for ultrasonic monitoring of subtle variations in forearm muscle thickness to achieve high-accuracy fine-grained gesture recognition, as it offers an optimal balance between spatial resolution and tissue penetration depth. Although higher frequencies can provide improved resolution, their significant attenuation in soft tissues hinders effective detection of deep muscle groups. Conversely, lower frequencies possess stronger penetration capability but suffer from reduced resolution, making it difficult to accurately track dynamic changes in thin tissue layers.

**Fig. 3.**
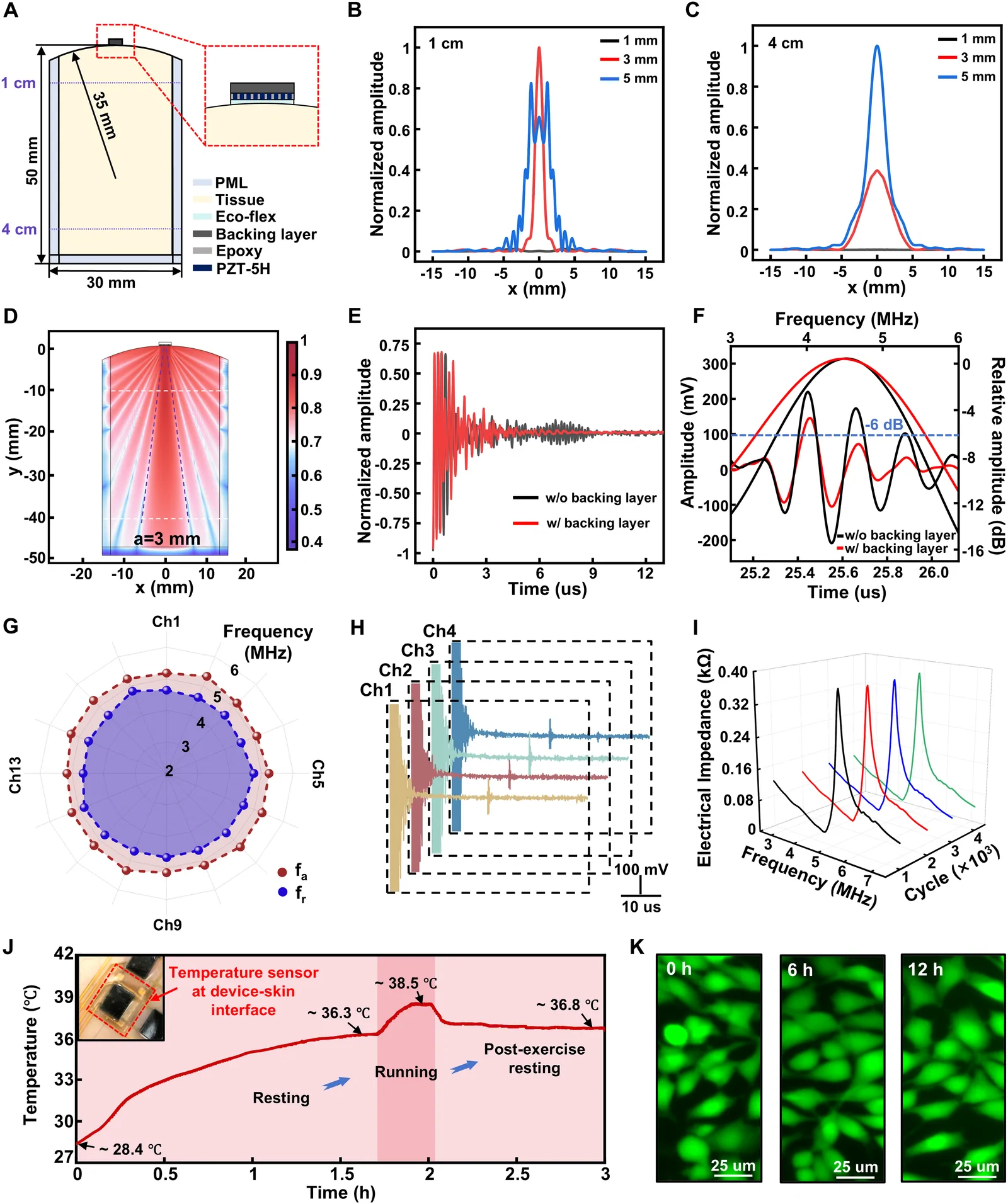
Performance characterizations of the stretchable multichannel ultrasonic device. (**A**) Schematic of the finite element model. Acoustic field profiles of the piezoelectric module of different widths (**B**) at the depth of 1 cm and (**C**) at the depth of 4 cm. (**D**) Simulated acoustic fields with the piezoelectric module width of 3 mm. (**E**) The ring-down time and (**F**) Pulse-echo response and frequency spectra of the piezoelectric module. (**G**) The resonance and antiresonance frequency variations of the 16 piezoelectric modules. (**H**) The received signals of the first four channels. (**I**) Electrical impedances of one typical channel under 30% tensile strain and cyclic loading for 1000, 2000, 3000 and 4000 times, showing the mechanical stability of the device. (**J**) Temperature variation curve of the device-skin interface under the condition of wearing a down jacket (simulating an environment with limited heat dissipation in winter). The inset in the top-left corner shows the thin-film temperature sensor positioned at the device–skin interface. (**K**) Fluorescence images of mouse fibroblast under ultrasound stimulation from the stretchable multichannel ultrasonic device for 0 hours, 6 hours, and 12 hours, respectively.

To obtain the optimal geometric parameters of the device for forearm muscle monitoring, we investigated the effect of the in-plane dimensions of the piezoelectric module on the ultrasonic wave propagation in the forearm using FEA. Additional details regarding the finite element simulations are provided in the Materials and Methods section. Figure 3A presents a schematic diagram of the finite element model with labeled components and key dimensional parameters. The model mainly consists of three parts: the piezoelectric module, the Eco-flex substrate layer, and biological tissue. To eliminate the influence of boundary reflection effects, a 2-mm-wide perfectly matched layer (PML) was placed around the biological tissue. The piezoelectric module was excited at a frequency of 5 MHz with an excitation voltage of 50 V. Given that forearm muscles are typically located at depths between 1 cm and 4 cm beneath the skin (*37*), it is preferable for the ultrasonic beam to maintain a relatively focused profile within this depth range. Figure 3, B and C show the acoustic intensity distributions at depths of 1 cm and 4 cm for piezoelectric module widths of 1 mm, 3 mm, and 5 mm, respectively. Combined with the simulated acoustic field distributions in Fig. 3D and fig. S7, it can be observed that a smaller module width (1 mm) results in main energy deviates from the center at 1 cm depth, causing significant energy dispersion, while at 4 cm depth the remaining energy of the ultrasonic beam is insufficient to perform high-quality monitoring. A larger module width (5 mm) leads to an extended near-field length, which in turn induces significant energy fluctuations that are unfavorable for ultrasonic detection. In contrast, a module width of 3 mm produces a well-focused beam with relatively high acoustic intensity at both 1 cm and 4 cm depths. We further investigated the effect of backing layer thickness on ultrasonic propagation (fig. S8). Based on these results, a piezoelectric module with effective working dimensions of 3 mm by 3 mm and backing layer thickness of 0.5 mm was selected for subsequent investigations.

Ultrasonic transmission and sensing rely on the reversible conversion between mechanical and electrical energy, making electromechanical performance a key metric for evaluating ultrasonic transducers. This performance mainly depends on two factors: i) the adoption of a piezoelectric layer with high coupling capability, and ii) the incorporation of a backing layer into the device architecture. The 1–3 anisotropic piezoelectric composite was selected as the active component of the device due to its exceptional electromechanical coupling performance. Compared to traditional PZT-5H, the epoxy resin fillers within the composite suppress the shear vibration modes, thereby enhancing the longitudinal coupling coefficient and improving the longitudinal ultrasonic wave propagation. The impedance and phase angle spectra of the 1–3 piezoelectric composite is shown in fig. S9. Meanwhile, it reduces the overall acoustic impedance (to ∼15 Mrayl), facilitating better acoustic coupling with soft tissues. A 35-mm-thick acrylic plate was used to evaluate the piezoelectric module, with detailed methods described in the Materials and Methods section. As shown in Fig. 3E, the ringing time of the device is reduced from ∼9 us without backing to ∼6 us with backing, corresponding to a 33.3% reduction that significantly enhances near-field detection capability. Figure 3F and fig. S10 present the measured pulse-echo response and its frequency spectrum. The backed device achieves a broad bandwidth (49.23%) and high longitudinal resolution (461 μm), representing improvements of 25.0% and 29.5%, respectively, over unbacked version (bandwidth: 39.37%, resolution: 654 μm). The device exhibits excellent channel consistency and operational stability. Impedance measurements of each piezoelectric module in the stretchable multichannel ultrasonic device (Fig. 3G) reveal the average resonant and anti-resonant frequencies of 4.67 MHz and 5.23 MHz, with standard deviations of 77.4 kHz and 72.8 kHz, across all 16 modules. Figure 3H presents the received signals from channels 1 to 4, while fig. S11 shows those from channels 5 to 16. Statistical analysis of these signals reveals mean ± standard deviation values of 6.32 μs ± 0.21 μs for ringing time, 1.43 μs ±0.19 μs for echo duration, and 198.4 mV ± 33.1 mV for echo amplitude, demonstrating high manufacturing reliability and channel uniformity. As shown in Fig. 3I, the electrical impedance of a typical channel remains stable after being subjected to a 30% tensile strain over 1000 to 4000 cycles. Furthermore, the echo signals remain almost unchanged even after 24 hours of continuous excitation (fig. S12).

The durability of the device is also closely related to the appropriate choice of coupling agents. As shown in fig. S13, the Derma-Tac pressure-sensitive adhesive was selected to replace conventional ultrasonic coupling gel. This silicone-based material exhibits acoustic impedance similar to that of both the substrate layer and the skin and achieves acoustic transmission performance comparable to conventional gel, while eliminating signal distortion caused by moisture evaporation. Moreover, it ensures reliable adhesion at the device-skin interface, significantly contributing to the long-term operation stability of the device. These results fully demonstrate the exceptional durability of the device.

Ultrasound induces biological effects through both thermal and mechanical mechanisms. It is therefore crucial to prevent thermal injury and mechanical damage during device operation to avoid discomfort or harm to the skin and underlying tissues. As illustrated in fig. S14 and Fig. 3J, the 3-hour surface temperature measurement of the device and a continuous 3-hour simulated real-world wearable scenario with heat dissipation constraints demonstrated its excellent thermal stability and superior thermal safety (note S5). To further evaluate the biocompatibility of device, cell experiments were performed using mouse fibroblasts (C2C12 cells) with the experimental setup schematically illustrated in fig. S15. Cells were stimulated in batches for 3, 6, 9, and 12 hours (see Materials and Methods for details). Representative results at pre-stimulation, 6 hours, and 12 hours post-stimulation are presented. The cell fluorescence images in Fig. 3K show that all cells remained viable, demonstrating the device’s excellent biocompatibility. In addition, fig. S16 shows that 24 hours of continuous wear did not cause visible skin indentations, moisture accumulation, allergic reactions, or inflammatory signs.

### Multichannel ultrasonic signals from fine-grained gestures

**Fig. 4.**
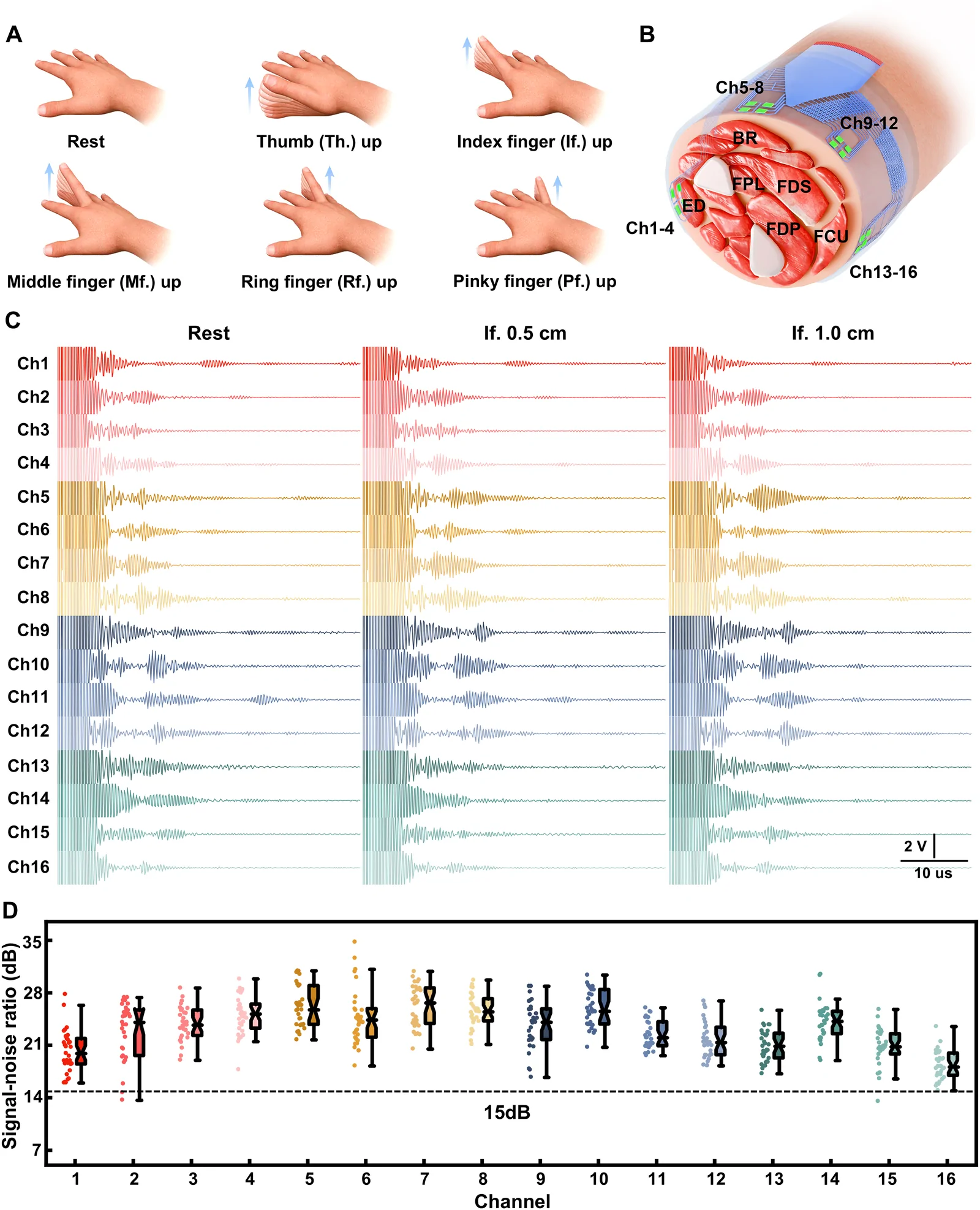
Display and comparative analysis of 16-channel ultrasonic signals in fine-grained gesture recognition tasks. The illustrations are created by graphics software Blender and Adobe Photoshop. (**A**) Schematic diagram of the proposed 31 fine-grained gestures. (**B**) The layout of the 16 channels and their spatial correspondence with the target muscles. (**C**) Representative 16-channel ultrasonic signals of three gestures (at rest, raise index finger by 0.5 cm and by 1 cm) collected from the forearm. Only the first 40 μs of representative A-mode ultrasound are shown to highlight the region with pronounced signal differences. (**D**) The signal-to-noise ratio statistical chart of the 16 channels for all the 31 gestures. Data are presented as mean ± SD across 31 gesture classes.

To demonstrate the capability of the stretchable multichannel ultrasonic device in distinguishing fine-grained multi-level gestures, we designed a set of finger movements with subtle kinematic differences (Fig. 4A). The gesture set comprised 31 actions: one static rest state and sequential lifting of each finger from 0 cm to 3.0 cm in 0.5 cm increments. Based on estimated average finger lengths, the corresponding metacarpophalangeal joint angles varied between 3.2° and 4.9°. This paradigm was chosen to provide a controlled benchmark for small-amplitude multi-level command recognition, because it enables graded finger motion while keeping the palm and wrist relatively stable. Compared with hand closing or finger bending, it reduces confounding effects from multi-joint coupling, palm deformation and wrist compensation, and is equipped with simple and stable quantitative standards at the same time (fig. S17).

These small angular changes effectively challenge the resolution limits of ultrasonic-based gesture recognition. The brachioradialis (BR), flexor digitorum superficialis (FDS), flexor pollicis longus (FPL), flexor digitorum profundus (FDP), flexor carpi ulnaris (FCU), and extensor digitorum (ED) were chosen as target muscle groups for gesture recognition due to their comprehensive and complementary roles in hand and forearm motor control. This muscle combination provides a robust physiological basis for decoding 31 gestures with subtle differences, including the rest and graded, individual finger lifts. Specifically, the FDS and FDP work synergistically to capture activation patterns related to the index, middle, ring and little fingers under varying flexion degrees. The FPL, as the primary flexor of the thumb, ensures unique kinematic signatures during thumb movement. The ED contributes to extension-related activity, creating an antagonistic contrast with flexor signals that helps in distinguishing motion initiation from rest. Furthermore, the inclusion of the FCU and BR enhances the detection of wrist rotation and forearm pronation/supination states, while suppressing interference from compensatory wrist motions during isolated finger tasks. By simultaneously monitoring the flexor, extensor, and stabilizer muscles, the stretchable multichannel ultrasonic device captures comprehensive neuromuscular information, greatly improving the accuracy and feasibility of fine-grained gesture recognition. The four functional sites of the stretchable multichannel ultrasonic device were aligned with the mid-forearm surface projections of these muscles, as illustrated in Fig. 4B, showing the 16-channel layout and their spatial correspondence with the target musculature.

To illustrate the performance of this 16-channel sensing system in distinguishing subtle kinematic differences, Fig. 4C presents signals during three representative gestures: rest, 0.5 cm index finger lift, and 1.0 cm index finger lift. This selection enables a comparative analysis of channel responses under minimal muscle activation and joint angle variations, highlighting the system’s high sensitivity and resolution in capturing fine-grained movement outputs. Even for a metacarpophalangeal joint angle change as small as ∼3.6°, notable differences can be observed in the ultrasonic signals across most channels, demonstrating the excellent ability of the device to recognize subtle movements. In some channels (e.g., Ch3, Ch8), signal variations under specific gesture are not significant, reflecting the inherent spatial heterogeneity of muscular deformation. Importantly, the channels that exhibit significant signal changes vary across different gestures, indicating that the multichannel configuration can capture complementary neuromuscular information. This spatial diversity highlights the advantages of the stretchable multichannel ultrasonic device: even when certain channels show limited responsiveness to a particular gesture, integration and collaborative analysis of all channels can compensate for individual limitations, thereby improving overall recognition accuracy and discriminative performance.

Figure 4D summarizes the signal-to-noise ratio (SNR) statistics across all 16 channels for each of the 31 gestures. Among the total 496 SNR measurements (16 channels × 31 gestures), only three fell below the 15 dB threshold (*33*), indicating that 99.4% of channel-gesture combinations exhibited signal quality well above the acceptable level. These results demonstrate the high signal sensitivity, excellent overall performance and good reliability of the stretchable multichannel ultrasonic device.

### Deep learning-assisted high-accuracy fine-grained gesture recognition

**Fig. 5.**
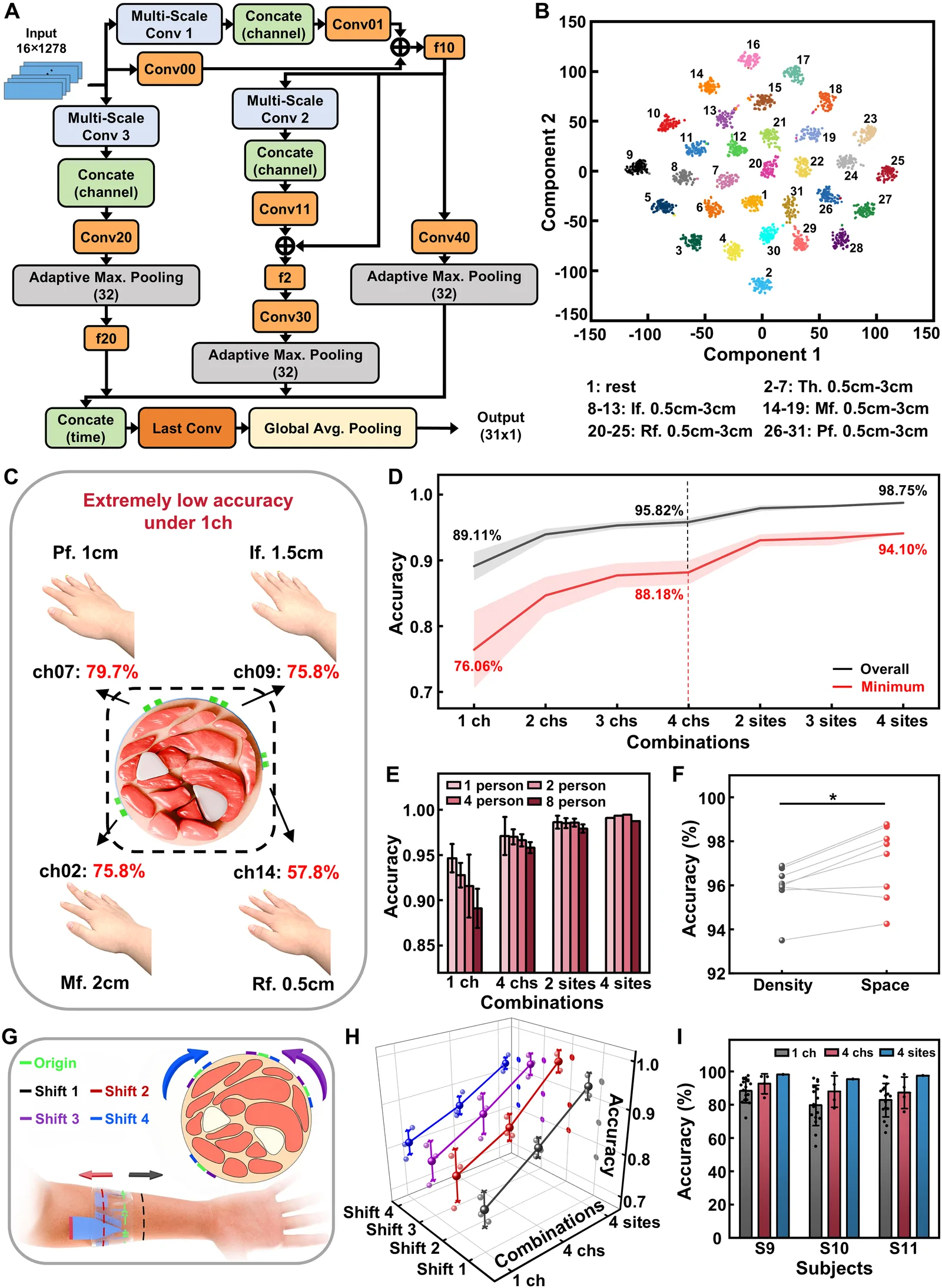
Deep learning assisted recognition of the fine-grained gestures. The illustrations are created by graphics software Blender and Adobe Photoshop. (**A**) Schematic flow chart of the 1D-CNN deep learning algorithm. (**B**) The cluster distribution for 31 gestures after dimensionality reduction using t-SNE. (**C**) The inherent limitation of single-channel configuration: inconsistent and poor recognition accuracy for specific gestures. (**D**) Effect of channel combinations on the recognition accuracy of fine-grained gestures. (**E**) Recognition accuracy of pooled-subject models trained using data from different numbers of participants. (**F**) Comparison between local-density and spatial-distribution four-channel selection strategies at subject-level. * indicates a statistically significant difference (*P* < 0.05), *n* = 8. Calibration-assisted recognition performance under shifted sensor positions, (**G**) Schematic of the device-placement protocol, including the original optimal position and four shifted positions, (**H**) Recognition accuracy of the 1 ch, 4 chs, and 4 sites configurations under the four shifted sensor positions after lightweight calibration. Light symbols indicate subject-level averaged values for each shifted position and channel configuration, with “1 ch”, “4 chs” and “4 sites” values averaged across all corresponding channel configurations for each subject. Dark symbols and error bars indicate mean ± SD calculated from these subject-level averaged values across three participants. Individual-subject statistics are shown in fig. S23. (**I**) Calibration-assisted recognition performance in three newly recruited participants. Data in [(D), (E) and (I)] are presented as mean ± SD. The 1 ch, 2 chs, 3 chs, 4 chs, 2 sites, 3 sites, 4 sites results were calculated across 16 single-channel selections, 24 two-channels selections, 16 three-channels selections, 4 four-channels selections, 6 two-sites selections, 4 three-sites selections and 1 four-sites selection respectively. The selections were treated as technical replicates.

To achieve high-accuracy recognition of 31 fine-grained gestures, we adopted a deep learning framework tailored for ultrasound monitoring, based on a multi-branch one-dimensional convolutional neural network (1D-CNN) architecture (*12*) (Fig. 5A). This network was improved to leverage the spatiotemporal characteristics of multichannel ultrasonic signals through an optimized multi-scale receptive field. By employing convolutional kernels sizes of 1, 3, 5, 7, and 11 in parallel in each multi-scale conv, the network captures feature patterns across multiple temporal scales. Smaller kernels perform fine scanning of the high-sampling-rate ultrasonic signals, enabling precise extraction of transient, high-frequency features associated with subtle muscle and tendon deformations while preserving the temporal resolution required to distinguish nuanced gesture differences. In contrast, larger kernels capture contextual patterns over extended periods. This multi-scale perceptual scheme, emphasizing fine details while incorporating medium and long-range patterns, provides a comprehensive representation of gesture dynamics, from brief twitches to sustained postures. The model uses multiple convolutional modules (Conv1, Conv2, etc.) for feature extraction, incorporates residual connections to improve gradient flow during training, and employs adaptive pooling to accommodate minor temporal variations in the acquired ultrasound frames. Performance monitoring and early stopping based on validation metrics are used to reduce overfitting during training. Combining this advanced neural network design with rigorous validation strategies, the proposed framework achieves high-accuracy recognition of subtle gesture variations, demonstrating the promising synergy between ultrasonic sensing and deep learning for human–machine interaction.

The stretchable multichannel ultrasonic device combined with the deep-learning framework was utilized to recognize the fine-grained gestures. A total of 8 healthy participants (3 females and 5 males, aged 25 ± 3 years) were recruited for ultrasonic signal collection (see Materials and Methods for details). The study primarily evaluates static graded finger gestures under stable attachment conditions and relatively consistent forearm postures. 6200 frames of data were collected per participant, and each frame contained 1278 sampling points for each of the 16 channels. Detailed settings are provided in the Materials and Methods section. Figure S18 presents the classification performance of the model for 31 categories of fine-grained gestures through a confusion matrix. The high values (close to 100%) along the diagonal indicate a large portion of correctly classified samples, achieving an overall accuracy of 98.75%. The near-zero-off-diagonal entries further confirm the strong discriminative power of the learned features and excellent inter-class separability. As shown in fig. S19, the training process is characterized by monotonic increases in accuracy (black curve) and continuous decreases in loss (red curve), with both metrics converging to a stable plateau. This behavior indicates that the model effectively learned discriminative features from the training data, maintained stability throughout the training process, providing a reliable basis for high-accuracy fine-grained gesture recognition. In Fig. 5B, t-distributed Stochastic Neighbor Embedding (t-SNE) is used to visualize high-dimensional ultrasonic features in two dimensions. These features are extracted from the last convolutional layer of the model, and after applying global average pooling along the temporal dimension, they are used for t-SNE visualization. The resulting plot reveals compact and well-separated clusters for different gesture categories, offering qualitative evidence of the model’s excellent discriminative capability and sensitive to subtle muscle deformations. Compared with previous studies on ultrasonic discrete gesture recognition and mainstream sEMG-based gesture-recognition systems, the proposed system distinguishes much smaller joint-angle changes while maintaining high recognition accuracy, highlighting its advantage for fine-grained gesture recognition (table S4).

The excellent recognition performance described above not only benefits from advanced algorithms but, more importantly, stems from the rich information provided by multichannel data. To quantify this advantage, we systematically investigate the relationship between the number of channels and recognition accuracy. When using single-channel data for deep learning, the recognition accuracy is quite low for specific gestures. As shown in Fig. 5C, Channel 2 achieves only 75.8% accuracy in recognizing the middle finger lifted by 2 cm, Channel 7 achieves 79.7% for the little finger lifted by 1 cm, Channel 9 achieves 75.8% for the index finger lifted by 1.5 cm, and Channel 14 performs poorly with a recognition accuracy of just 57.8% for the ring finger lifted by 0.5 cm. These results indicate that single-channel data has significant limitations in comprehensively recognizing all gestures. To improve the recognition task, it is essential to study the impact of incorporating multiple channels. With the configuration of 16 channels across four functional sites, we statistically analyzed the recognition accuracy of the following combinations: one channel (1 ch), two-channel (2 chs), three-channel (3 chs), four-channel (4 chs) combinations within a single site, as well as two-site (2 sites), three-site (3 sites), and four-site (4 sites, all 16 channels) combinations. Figure 5D shows that as the number of channels increases, the overall recognition accuracy increases from 89.11% to 98.75% (a growth rate of 10.82%), and the minimum recognition accuracy among the 31 gesture categories increases from 76.06% to 94.10% (a growth rate of 23.72%). This indicates that multi-channel configurations not only improve the average performance but, more importantly, enhance the worst-case accuracy, ensuring consistent high-accuracy recognition for all gesture categories, which is crucial for practical applications.

We also performed a statistical overview of all gestures to investigate the impact of number of channels. As shown in fig. S20, as the number of channels increases, the number of gestures (out of 31) with recognition accuracy below 90% gradually decreases, and a two-site channel combination is sufficient to completely eliminate accuracies below 90%. Concurrently, the number of gestures with recognition accuracy above 95% gradually increases, and when all 16 channels are used, 30 gestures achieve recognition accuracy exceeding 95%. These results demonstrate that multichannel information fusion not only improves recognition accuracy but also effectively mitigates the “long-tail problem” typical in classification tasks, where a minority of challenging movements impair overall system performance. By eliminating low-accuracy outliers and shifting the majority of gestures into the high-accuracy saturation region, the multichannel approach enables a qualitative leap from mostly-usable to fully-usable performance, thereby laying a solid foundation for practical applications.

Furthermore, tables S5 and S6 present recognition results for two representative movements of index finger lifted by 3.0 cm and ring finger lifted by 1.5 cm using channel combinations within functional site 2 and across all four functional sites. Extending this analysis to all channel combinations and all 31 movements, we found that no single combination alone can simultaneously achieve the highest accuracy across all gestures. For example, the two-channel combination of ch9 and ch10 yields the highest accuracy (96.50%) for ring finger lifted by 1.5 cm among all dual-channel combinations, but only reaches 88.30% for index finger lifted by 3.0 cm. The latter movement achieves its highest accuracy (90.20%) with the combination of ch9 and ch11. Only when all four channels within a single site or all four functional sites are used, the optimal performance can be achieved across all movements.

In addition to improving class-wise recognition performance for fine-grained gestures, the multichannel approach is also more effective in learning of shared gesture-discriminative features from heterogeneous multi-subject data. As shown in Fig. 5E, when the number of participants increases from 1 to 8, the recognition accuracy of the single-channel configuration exhibits a noticeable decline, dropping from 94.65% to 89.11%. As the number of channels increases, this performance drop gradually diminishes. Under the “4 sites” configuration, the system’s recognition accuracy remains at a high level with minimal fluctuations across different user scales (99.16% ± 0.31%). These results indicate that the richer information provided by the multichannel device helps the model accommodate inter-subject variations in muscle morphology, tissue deformation, and movement patterns, thereby maintaining relatively stable recognition performance as the pooled-subject dataset becomes more heterogeneous.

Having confirmed the advantages of the multichannel configuration, we next investigated whether its performance gain mainly originated from increased channel density within a local sensing site or from spatial diversity across different sensing sites. To this end, we compared two four-channel strategies: a “density” strategy, in which four channels were selected from the same functional site, and a “space” strategy, in which four channels were selected from different functional sites. To purely compare and analyze the effects of single-site sensor density and spatial diversity of muscles, in the “space” strategy, we selected channels at identical relative positions from each of the four functional sites to completely eliminate spatial variations within individual sites. As shown in Fig. 5F and table S7, after averaging different four-channel combinations within each strategy for each subject, subject-level paired analysis showed that the space strategy (97.06 ± 1.66%) achieved significantly higher recognition accuracy than the density strategy (95.92 ± 1.06%), with a 95% confidence interval of 0.38% to 1.90% for the paired difference and p = 0.023 by the Wilcoxon signed-rank test. These results indicate that the benefit of multichannel sensing is not merely due to increasing channel density within a local region. Instead, distributing channels across different functional sites provides more diverse and complementary gesture-related spatial information, which plays a more important role in improving fine-grained gesture recognition.

In practical applications, recognition performance may decrease when the device is reattached in a new session or applied to an unseen user, owing to changes in sensor-skin coupling, sensor placement, muscle morphology, and movement patterns (figs. S21 and S22). To address this issue, we introduced a lightweight calibration strategy for new sessions and new users. When a new session or user was introduced, a small amount of calibration data was collected, and the pre-trained model was rapidly adapted using only 10 calibration frames (only 1 second for acquisition) for each gesture class. During calibration, most parameters of the pre-trained backbone network were frozen, while only the last two key convolutional layers (Conv30 and Conv40) were partially unfrozen. An adaptive calibration layer was further incorporated to adjust the feature distribution to the new session or user without retraining the entire backbone network. Detailed model training hyperparameters are listed in table S8.

As shown in Fig. 5G, the device was shifted by 1 cm toward the wrist, elbow, radial side, and ulnar side, defined as Shift 1, Shift 2, Shift 3, and Shift 4, respectively. After lightweight calibration, the model maintained high recognition performance across these four shifted device positions in three participants randomly selected from the original cohort of eight participants (Fig. 5H). Using the full “4 sites” configuration, the recognition accuracies under Shift 1–4 were 93.92 ± 2.85%, 97.07 ± 2.43%, 94.40 ± 2.46%, and 92.58 ± 1.39%, respectively. These results indicate that the calibration strategy effectively compensated for performance degradation caused by session-specific placement offsets. In the unseen-user test, the calibrated model also achieved stable recognition performance in three newly recruited participants, with accuracies of 98.22%, 95.44%, and 97.55% for S9, S10, and S11, respectively (Fig. 5I). The raw data before and after calibration are provided in table S9. Compared with the “1 ch” and “4 chs” configurations, the full “4 sites” configuration consistently achieved higher average recognition accuracy and more balanced performance across all tested conditions (all above 90%), without an apparent weak condition. These results demonstrate that the richer information provided by the multichannel device enables more effective calibration-assisted adaptation to both shifted device positions and new users.

### Demonstration of non-verbal communication enabled by subtle graded gestures

To further demonstrate the practical applicability of the proposed ultrasonic device for real-time human-machine interaction, we have created a non-verbal communication interface as a representative application scenario. The experiment was conducted with a relatively stable forearm posture. This interface is designed for patients who have difficulty expressing their needs verbally or through conventional movements, such as individuals with aphasia, postoperative intubation, dysphagia, or impaired mobility. In this demonstration, small-amplitude graded finger lifting was mapped to predefined communication commands: relax indicated no demand. Thumb lifting by 1, 2, and 3 cm represented mild respiratory discomfort, respiratory distress, and severe respiratory distress, respectively. Index finger lifting by 1, 2, and 3 cm represented mild, moderate, and severe pain, respectively. And ring finger lifting by 2 cm represented emergency call. The corresponding demonstration is shown in movie S1.

During real-time inference, each complete 16-channel ultrasonic frame was independently processed by the trained 1D-CNN model to generate one prediction. A command was output only when two consecutive predictions produced the same predicted class and both frames had maximum softmax confidence values exceeding 50%. Command-level performance was further summarized in table S10. For the non-verbal assistive communication scenario, an attempt was considered successful only when the first command output generated by the interface within 1 s matched the intended command. The single-frame model inference time was consistently below 3 ms, and the overall end-to-end delay across all tested commands was 227.5 ± 140.8 ms. The command-level results showed that the “no demand” state and “emergency call” command both achieved a 100% correct rate, indicating reliable recognition of the idle and urgent communication states. The graded pain-related commands achieved correct rates of 75.0%, 75.0%, and 87.5% for mild, moderate, and severe pain, respectively, while mild and severe respiratory distress achieved correct rates of 87.5% and 75.0%, respectively. Incorrect outputs mainly occurred between neighboring levels or between the lowest-amplitude command and the rest state, reflecting the intrinsic challenge of distinguishing fine-grained multi-level gesture states during real-time operation. Nevertheless, the sub-second response time, millisecond-level model inference, and overall favorable command-level performance demonstrate the feasibility of using subtle graded finger movements for low-burden assistive communication.

## DISCUSSION

In this work, we propose a stretchable multichannel ultrasonic device for fine-grained multi-level gesture recognition. The device consists of several key components: sixteen single-sided welded piezoelectric modules made of 1–3 piezoelectric composite material with a backing layer, an electrical interconnection based on a sandwich-style serpentine film, a flexible Eco-flex substrate, a flexible Eco-flex encapsulation, and a Derma-Tac silicone adhesive layer for skin attachment. Featuring a unique fan-shaped design, the device can be stretched up to 30%, enabling conformal forearm attachment and alignment with target muscle regions. With the detection depth preset to 4.92 cm, it is shown that the proposed device achieves a sub-millimeter spatial resolution of 461 μm and an excellent signal-to-noise ratio (99.4% of the signals exceeding 15 dB). When integrated with a one-dimensional convolutional neural network suitable for ultrasonic signal processing, the proposed system can achieve a recognition accuracy of up to 98.75% for subtle angular variations ranging from 3.2° to 4.9° in the metacarpophalangeal joints, which verifies the recognition capability of ultrasound equipment for tiny joint movements. The multichannel configuration yields high classification accuracy, improved class-wise recognition balance, more effective learning from multi-subject data and enhanced calibration-assisted adaptation to shifted device positions and new users, providing a stretchable multichannel ultrasonic strategy for reliable, low-burden, fine-grained multi-level gesture command control.

Future research will focus on four key aspects. First, to address broader human-machine interaction applications, we will extend the experimental paradigm to more representative hand movements, such as hand closing and finger bending, and integrate devices such as optical tracking systems and strain-sensing gloves to enable continuous joint-angle prediction and multi-finger motion decoupling. Second, we will expand the scale of participants and include diverse populations (different ages, BMI and exercise capacity) to improve the generalization ability of the system in real-world applications. Third, to address the issue of recognition stability under dynamic conditions, at the hardware level, non-target interference can be minimized in the future through the selection of more stable conformal fixation structures and sensing functional areas. At the algorithm level, future work will introduce more targeted dynamic robustness enhancement strategies, such as dynamic data augmentation methods including noise perturbation, time translation, amplitude scaling, and random channel dropout to improve the generalization ability and real-time recognition stability of the model under non-stationary conditions. Output stability could be further improved while maintaining real-time responsiveness by incorporating signal-processing or algorithmic correction modules to suppress dynamic artifacts. In addition, a higher ultrasonic pulse repetition frequency would allow a larger temporal voting window to be used for inference results without substantially increasing response latency. Fourth, we will develop fully integrated stretchable multichannel ultrasonic systems incorporating on-device signal processing, gesture recognition, and wireless communication to improve portability and support long-term dynamic health monitoring.

## MATERIALS AND METHODS

### Fabrication of stretchable multichannel ultrasonic device

Figure S3 shows the schematic diagram of the fabrication process for the stretchable multichannel ultrasonic device. A uniform layer of epoxy resin (Epo-tek 301, Epoxy Technology, USA) was applied on the surface of the 1–3 piezoelectric composite material (Baoding Xinwei Electronic Technology Co., Ltd., China). The backing layer (Hunan Xiongwu Technology Co., Ltd., China) was adhered to the 1–3 piezoelectric composite material, and a firm pressure was applied to the bonded area. The assembly was then cured in an oven at 65°C for 1 hour to complete the fabrication of the piezoelectric module. A ∼50 μm thick Eco-flex substrate layer (Eco-flex 00–30, Smooth-On, USA, cured at room temperature for 4 hours) was doctor-bladed on a glass plate coated with a mold release agent (RD-518) using a square doctor blade. The stretchable circuit with a sandwich structure fabricated by the flexible printed circuit (FPC) process was transferred onto the Eco-flex substrate layer. The contact surface was pre-treated with UV ozone for 10 minutes to enhance adhesion. Subsequently, the piezoelectric module and the stretchable circuit were connected using low-temperature solder paste (KZ-1513, KELLYSHUN, China). The soldering was completed by heating on a hot plate set at 150°C for 3 minutes. Another layer of Eco-flex packaging material was then covered and cured (Eco-flex 0030, Smooth-On, USA, ∼1.05 mm, cured at room temperature for 4 hours). The device was peeled off from the glass plate, and a ∼13 μm thick silicone-based pressure-sensitive adhesive (Derma-Tac, Smooth-On, USA, cured at room temperature for 10 minutes) was doctor-bladed on the surface of the substrate layer, thereby completing the fabrication of the stretchable multichannel ultrasonic device. The sources of all major materials for the device are summarized in table S1.

### Finite element simulation of stretchable serpentine wire deformation

Finite element analysis was performed to study the deformation of stretchable serpentine wire using the commercial software ABAQUS. The encapsulation layer was modeled as a hyperelastic material and described by the Mooney–Rivlin model with parameters *C_10_* = 0.008 MPa, *C_01_* = 0.002 MPa, and *D1* = 2 MPa^−1^. Polyimide (PI) was defined as a linear elastic material with Young’s modulus of 2.5 GPa and Poisson’s ratio of 0.34. Copper (Cu) was modeled as an ideal elastic–plastic material with Young’s modulus of 124 GPa, Poisson’s ratio of 0.33, and a yield stress of 372 MPa. C3D8R hexahedral elements were assigned to Cu and PI, while C3D8RH hexahedral elements were employed for the flexible encapsulation material (see fig. S24 for details of the model).

### Finite element simulation of ultrasound propagation

To investigate the effect of the piezoelectric module width on ultrasound propagation in the forearm, finite element analysis was performed using COMSOL software. The 3D structures of the piezoelectric module and the forearm were simplified into a 2D plane for simulation, as shown in Fig. 2A. The 1–3 piezoelectric composite material in the piezoelectric module has a thickness of 300 μm, the backing layer has a thickness of 500 μm, and the width of the piezoelectric module is the variable to be studied. The forearm was simplified to a circle with a radius of 35 mm, and this dimension was calculated based on the average circumference of the cross-section at the middle of the forearm from 13 adult participants (aged 25 ± 3 years, 10 males and 3 females). Meanwhile, to save computational resources and improve computational efficiency, the circle was partially truncated to obtain the final 2D planar model of the forearm. A substrate layer was placed between the forearm and the piezoelectric module, with a thickness of 50 μm and a width consistent with that of the piezoelectric module. It is reasonable to ignore the ultra-thin adhesive layer and the epoxy resin bonding layer, as their influence on ultrasound propagation is negligible. In the frequency-domain modeling, three physical fields—pressure acoustics (frequency domain), electrostatics, and solid mechanics—were selected, and a multiphysics coupling interface for acoustic-structural boundaries and piezoelectric effects was adopted. “Pressure acoustics (frequency domain)” acts on the Eco-flex layer and the biological tissue layer. In the biological tissue layer, all boundaries except the upper surface were treated with a 2 mm perfectly matched layer (PML). “Solid mechanics” acts on the backing layer, the epoxy resin, and PZT-5H. PZT-5H was set as a piezoelectric material, while all other materials were set as linear elastic materials. “Electrostatics” acts on PZT-5H only, an excitation voltage of 50 V was applied to its upper surface, and the bottom surface was grounded to 0 V. In the COMSOL simulation, built-in material parameters were used for PZT-5H and epoxy resin. For other required material parameters, see table S2 for details.

### Device performance characterization

The impedance and phase angle of the device were measured using an impedance analyzer (TH-2850), with a scanning frequency range of 2 MHz to 8 MHz and a step size of 0.03 MHz. The resonant frequency (*f_r_*) and anti-resonant frequency (*f_a_*) can be obtained from the impedance curve. All performance tests of the piezoelectric module were conducted using Verasonics ultrasound system, which adopted negative square wave pulse and provided an excitation with a frequency of 5 MHz and a peak voltage of −50 V. The test object was a 35 mm thick acrylic plate, and pulse-echo signals were received from its bottom interface. The bandwidth of the piezoelectric module is determined by the frequency bandwidth at −6 dB, which is calculated using the following formula: $\mathrm{BW}=\frac{f_{U}-f_{L}}{f_{C}}\times 100\%$, where *f_U_* represents the upper frequency, *f_L_* represents the lower frequency, and *f_C_* represents the center frequency. In this study, a fatigue testing machine (Model M-100, CARE Measurement & Control) was used to perform fatigue tests on the device. The tensile strain was set as 30% and the loading frequency was 1.0 Hz. After completing 1000, 2000, 3000, and 4000 cycles of loading, the impedance of one typical channel was measured using an impedance analyzer (TH-2850). The surface temperature of the device was calibrated using a thermal imager (C400, Guide, China). A thin-film temperature sensor (PT100, Sense Future, China) and a data acquisition/multimeter system (DAQ 6510, KEITHLEY, USA) are used to dynamically monitor the temperature changes at the device-skin interface. The thin-film temperature sensor is placed at the device-skin interface below a piezoelectric module for monitoring, wherein the resistance-temperature characteristic of the thin-film temperature sensor is *R*(*t*) = 100 × (1 + 0.00569 *t* + 7.19059 × 10^−6^ *t*^2^ + 3.44257 × 10^−11^ *t*^4^), where *t* stands for temperature, and *R*(*t*) is the function of resistance varying with temperature.

### Biocompatibility assessment

C2C12 mouse fibroblasts were purchased from the American Type Culture Collection (ATCC) and cultured in Dulbecco’s Modified Eagle’s Medium (DMEM, ATCC) supplemented with 10% fetal bovine serum (FBS, Gibco) and 1% penicillin/streptomycin (Gibco) in an incubator at 37°C with 5% CO_2_. After passage, C2C12 cells were seeded into 5 petri dishes (35 mm in diameter) at a density of 1 × 10^4^ cells/cm^2^, and cultured for another 24 hours until the cell confluency reached approximately 70%–80%. The control group was placed in the incubator, while the other 4 petri dishes were placed on the top of 4 functional sites. A signal generator (RIGOL DG952) and a power amplifier (Relaxation Ultrasound, China) were used to provide negative square wave pulse excitation with a frequency of 5 MHz, a peak voltage of −50 V, and a pulse repetition frequency of 40 Hz. After 0 (control group), 3 (functional site 1), 6 (functional site 2), 9 (functional site 3), and 12 (functional site 4) hours of ultrasound exposure, the cells were stained with calcein AM (Solarbio, final concentration of 2 μM) and propidium iodide (PI, Solarbio, final concentration of 5 μM) by incubation for 10 minutes in a 37°C, 5% CO_2_ incubator. Subsequently, imaging was performed using a live-cell imaging system (Observer Z1, Zeiss).

### Signal acquisition of 31 fine-grained hand gestures

In this study, 8 healthy participants from the university community (3 females and 5 males, aged 25 ± 3 years, see table S3 for details) were randomly recruited for ultrasonic signal collection. The sample size of eight participants was determined based on sample sizes commonly used in previous ultrasound gesture-recognition studies. The functional sites of the stretchable multichannel ultrasonic device were attached to the body surface projections of the target muscles in the middle of the forearm. The participants sat upright in front of a computer screen, with their palms placed flat on the table in a relaxed state. Their forearms were kept level with the table and perpendicular to the edge of it. According to the instructions, the participants were required to complete a total of 31 static gestures, including 1 resting gesture and 30 gestures where fingers were lifted upward (specifically, each of the 5 fingers was lifted sequentially from 0 cm to 3.0 cm, with an increment of 0.5 cm each time), motion calibration is completed by aligning the fingertip with the ruler scale, as shown in fig. S17. To meet the requirements of static gesture recognition, the Verasonics ultrasound system was configured as follows: negative square-wave pulses at 5 MHz with a peak amplitude of −50 V were applied in a 16-channel polling mode, where each polling cycle was defined as one group, repeated at 10 Hz, with 16 channels transmitting sequentially at equal intervals. For each echo acquisition cycle, 1278 sampling points were recorded at a 20 MHz sampling rate. Under these settings, the detection depth reached 49.2 mm, sufficient to cover the muscle–bone interface of the forearm. Each static gesture was maintained for 25 s, with a 15 s rest interval between gestures to avoid muscle fatigue. All experiments on human skin were approved by the Human Research Ethics Committee of Zhejiang University (approval no. 2025156). Informed consent was obtained from all participants.

### Signal preprocessing process

To eliminate transient effects during gesture transitions, only the last 20 s of each gesture were used for analysis, including 200 frames of ultrasound data. Prior to input into the neural network, raw ultrasonic signals were subjected to a standardized preprocessing workflow to enhance signal quality and feature discriminability, as illustrated in fig. S25. First, time gain compensation was applied to the signals to correct for ultrasonic attenuation caused by depth. Subsequently, a band-pass filter was implemented to retain effective frequency band components and suppress out-of-band noise. Then, the Hilbert transform was used to extract the envelope curve of the signals, highlighting the distribution characteristics of echo energy. Finally, logarithmic compression was employed to compress the dynamic range of the envelope signals, making them suitable for neural network processing while enhancing numerical stability and feature separability.

### Model implementation and evaluation

The 1D-CNN model was developed in Visual Studio Code (v. 1.125.1) and implemented in Python (v. 3.10.19) using the PyTorch deep-learning framework (v. 2.10.0, CUDA 13.0 build). Model training and calibration were performed on a Windows 11 workstation equipped with an AMD EPYC 9554 64-core processor and an NVIDIA RTX 3090 GPU. GPU acceleration was performed using the CUDA framework (v. 13.0). During the real-time human–machine interaction demonstration, model inference was performed on the CPU. In model training process, for each subject and each gesture class, the acquired ultrasonic frames were partitioned deterministically according to acquisition order. Specifically, the first 70% of the frames were used as the training set, the subsequent 15% were used as the validation set, and the final 15% were used as the test set. For the new-session and new-user evaluations, a small calibration dataset was acquired before testing. For each shifted device position or new user, 40 complete 16-channel ultrasonic frames were collected for each of the 31 gesture classes at the same 10 Hz polling-cycle rate. The first 10 frames were used for lightweight fine-tuning of the pre-trained model, corresponding to approximately 1 s of data acquisition per gesture class, whereas the remaining 30 non-overlapping frames were used only for testing. The reported recognition accuracy was obtained from a single training run. For the real-time human-machine interaction demonstration, the end-to-end delay was measured from recorded videos using a custom script and was defined as the time from stabilization of the target gesture to the first valid command output displayed by the interface.

## Supplementary Materials

### The PDF file includes:

Supplementary Text Figs. S1 to S25 Tables S1 to S11 Legend for movie S1 References
