# Supplementary material for: Fine-grained multi-level gesture recognition based on a stretchable multichannel ultrasonic device
Source: Sci Adv. 2026 Sep 23;12(39):eaef1101. doi: 10.1126/sciadv.aef1101 (PMC13600148; doi:10.1126/sciadv.aef1101)
Supplement: Supplementary file 1 [file sciadv.aef1101_sm.pdf]

Science Advances
AAAS

Supplementary Materials for

Fine-grained multi-level gesture recognition based on a stretchable multichannel ultrasonic device

Xinyi Lin *et al.*

Corresponding author: Jizhou Song, jzsong@zju.edu.cn

Sci. Adv. **12**, eaef1101 (2026)
DOI: 10.1126/sciadv.aef1101

The PDF file includes:

Supplementary Text
Figs. S1 to S25
Tables S1 to S11
Legend for movie S1
References

Other Supplementary Material for this manuscript includes the following:

Movie S1

Supplementary Text

Note S1. 4by4 layout of 16 piezoelectric modules.

Designing 16 piezoelectric modules into a 4×4 layout consisting of 4 functional sites with a 2×2 arrangement within each site is based on the following considerations:

First, as described in the section "Multichannel ultrasonic signals from fine-grained gestures", six key target muscle groups are adopted to achieve high-precision gesture recognition. Through carefully arranged four functional sites, the four functional sites can precisely cover these core muscle groups, ensuring comprehensive sampling and effective characterization of key motor units.

Second, in existing research, ultrasonic probes used for gesture recognition are mostly circular rigid probes with a diameter of 9-12 mm (*42, 44*), whose size and rigidity limit their fit with the skin. Adopting a 2×2 array layout within each functional site can achieve an effective coverage area of 9 mm×9.6 mm, which is similar to that of traditional probes, while rendering rigid probes flexible, significantly improving skin conformity and wearing comfort, thereby obtaining more stable and high-quality ultrasonic signals.

Note S2. Asymmetric serpentine interconnect structures in only three stretchable regions.

We adopted the asymmetric serpentine interconnect design only in three stretchable regions for the following considerations.

First, the sensing area does not prioritize stretchability but instead divides the original rigid probe into four smaller modules to achieve flexible adhesion rather than overall stretching. If the sensing area were stretchable, it would compromise positional stability during repeated device placement. Therefore, we intentionally avoided this design consideration.

Second, this design adopts an asymmetric serpentine interconnection layout, with the aim of taking functional site 2 as the core positioning reference. To securely anchor the reference point, the serpentine stretchable structure between functional site 2 and the flexible interconnection zone is eliminated, establishing a rigid positioning anchor that ensures positional stability and prevents drift during wear and stretching. Building upon this foundation, the flexible interconnection serves as a mechanical fulcrum, enabling adaptive attachment of remaining sites through unilateral stretching of serpentine conductors. This achieves mechanical decoupling between reference anchoring and site adjustability. The design maintains high positioning accuracy in the core measurement area while accommodating anatomical size variations among subjects, significantly improving positional consistency during repeated adhesions and signal measurement reliability. The combination of reference anchoring and mechanical fulcrum constitutes the critical design for achieving precise attachment.

Note S3. Perfectly matched layer.

Perfectly matched layer is a segmented boundary region of the biological tissue, which absorbs the ultrasonic energy reflected from the boundary during the simulation, simulates an infinite medium environment, thus improving the simulation accuracy. Its material is the same as that of biological tissue, both being "Tissue" in **Table S2**.

Note S4. Bandwidth comparison with commercial sensors and other wearable ultrasonic sensors.

First, the bandwidth of this device is lower than that of commercial rigid ultrasonic probes, mainly due to the following structural and design differences:

(1) The thickness of the backing layer is much smaller than that of commercial probes: commercial ultrasonic probes generally adopt thick high-damping backing layers, which can effectively suppress the ringing effect of piezoelectric elements, broaden the frequency response range, and thus achieve high bandwidth. To meet the requirements of thinness and flexibility for wearable applications, this device uses a thinner backing layer, which relatively prolongs the ringing time of the piezoelectric elements and directly restricts the expansion of bandwidth.

(2) No acoustic matching layer is provided: commercial probes are usually equipped with multiple acoustic matching layers to form a gradual impedance transition between piezoelectric materials and human tissue, ensuring efficient sound wave transmission over a wide frequency range. To simplify the structure and reduce the overall thickness, this device omits the matching layer, resulting in relatively large acoustic impedance mismatch at the interface. Energy transmission efficiency is only favorable near the center frequency, further limiting the bandwidth.

(3) Impact of miniaturized element size: commercial probes have relatively large element sizes with optimized structural parameters, leading to single and stable resonant modes. In this study, miniaturized elements are adopted to achieve a compact array layout and improve conformability to the human body. The small size introduces edge effects and parasitic resonances, degrading the frequency response characteristics and ultimately reducing the bandwidth.

(4) Additional losses introduced by flexible packaging: commercial probes use rigid packaging materials with stable acoustic properties, introducing almost no additional acoustic losses. To achieve good conformability with the skin, this device employs flexible polymer packaging. Such flexible materials introduce certain mechanical losses and acoustic attenuation, disrupting the ideal resonant state of the transducer and narrowing the bandwidth.

The adoption of a thin backing layer, omission of the matching layer, miniaturized elements, and flexible packaging in this study represents a necessary trade-off to achieve the core wearable characteristics of the device: miniaturization, flexibility, thinness, and easy skin adhesion. Although the above design reduces the bandwidth compared with commercial rigid probes, the performance degradation is fully within an acceptable range. The ultrasonic signals output by the

device still possesses sufficient signal-to-noise ratio and effective characteristic information, which can fully meet the application goal of fine gesture recognition. Meanwhile, the improved wearing adaptability and signal stability brought by flexible conformability are more critical for wearable gesture recognition tasks.

Second, even with the above trade-offs, the bandwidth performance of this device is superior to that of flexible ultrasonic sensors reported in existing literature that only use a backing layer and lack a matching layer (its relative bandwidth is comparable to similar studies, and the absolute bandwidth reaches 2.46 MHz, outperforming sensors with similar configurations). **Table S11** lists the bandwidth data of this device and representative similar flexible ultrasonic devices for detailed explanation and verification.

Note S5. Long-term thermal safety testing.

First, a surface temperature test of the device during 3 consecutive hours of operation was conducted. As shown in **Fig. S14**, monitoring via an infrared thermal imager revealed that the surface temperature of the device remained stable at 24.9±0.2 °C with no obvious temperature rise. This result indicates that due to the extremely short excitation duration of pulsed ultrasound, energy accumulation is negligible, and the device exhibits almost no self-heating during continuous operation, demonstrating favorable fundamental thermal stability.

To further simulate real wearing scenarios, we dynamically monitored the temperature changes at the device-skin interface using thin-film temperature sensors while wearing down jackets (to simulate a heat dissipation-restricted environment in winter), as shown in **Fig. 3J**. During daily working conditions, the interface temperature rose slowly with human metabolic heat production and reached a steady state of 36.3 °C after approximately 100 minutes. This resulted from a thermal equilibrium between human heat generation and restricted heat dissipation caused by clothing and the device. When switching to running at a cadence of 180 steps/min, metabolic heat production increased significantly, the temperature rise rate accelerated markedly, and the temperature stabilized at 38.5 °C after about 12 minutes of continuous exercise, demonstrating the superimposed effect of high-intensity exercise and a heat dissipation-restricted environment. After exercise, the interface temperature dropped rapidly first (due to a sharp decrease in metabolic heat production) and then slowly fell back to 36.8 °C and remained stable, a process reflecting the gradual dissipation of residual heat in the skin and the interface. The steady-state temperature after exercise was 0.5 °C higher than that before exercise, mainly attributed to residual heat retention in the interface region after strenuous exercise and the persistently restricted heat dissipation environment. It is worth emphasizing that throughout the testing cycle with extremely poor heat dissipation and accompanied by intense exercise, the device-skin interface temperature remained consistently below 39 °C, not reaching the threshold that may cause skin discomfort or safety risks.

These results fully demonstrate that the stretchable multi-channel ultrasonic device exhibits excellent thermal safety and biocompatibility under long-term continuous wearing and high-intensity usage scenarios, and can meet the reliability requirements for practical applications.

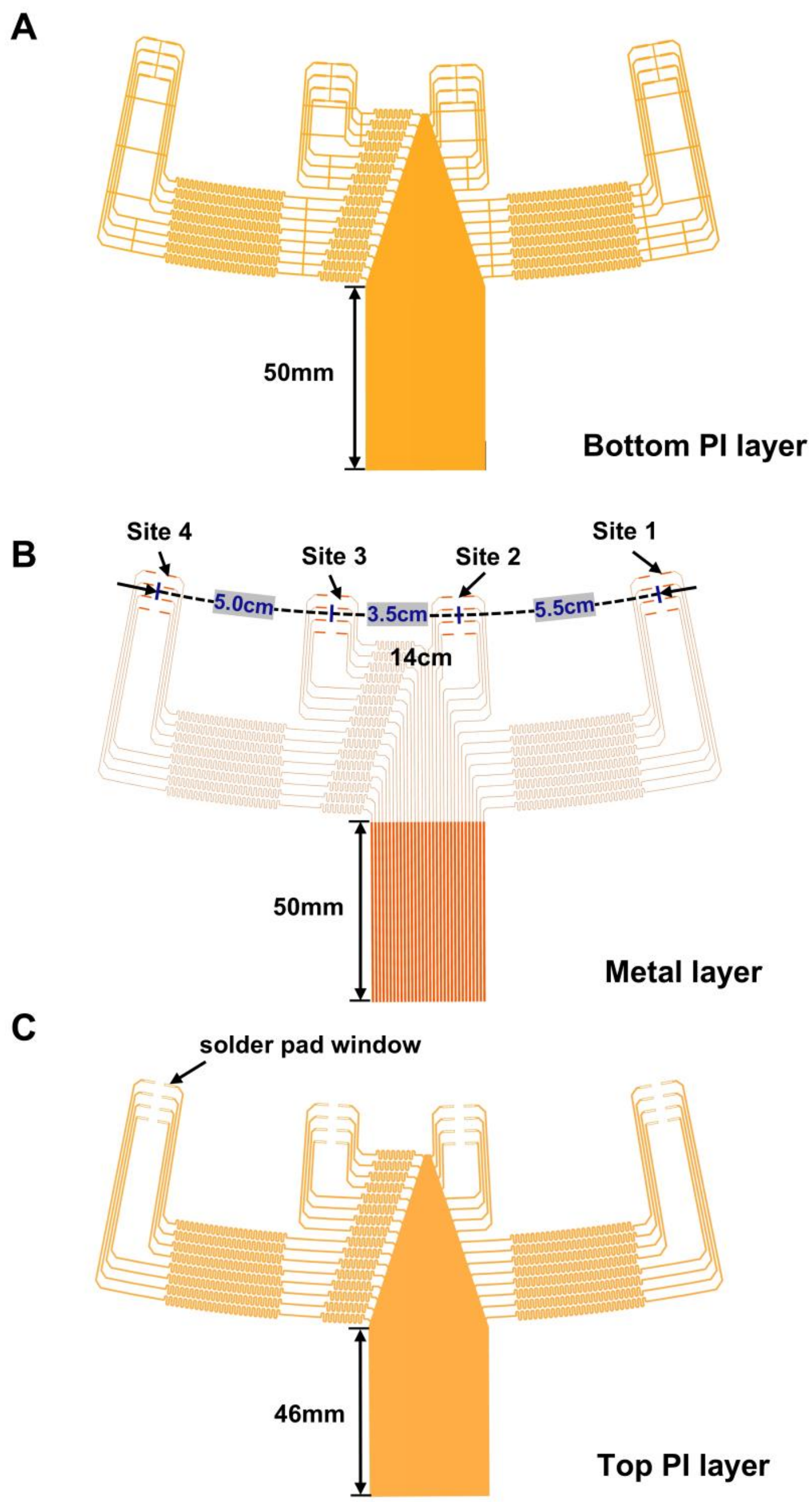

Fig. S1. Schematic diagram of stretchable circuit layer. (A) Schematic diagram of bottom PI layer. (B) Schematic diagram of metal layer. The arc length of the midline in the functional site area is 14 cm. Among them, the distance between the centers of site 1 and site 2 is 5.5 cm, the distance between the centers of site 2 and site 3 is 3.5 cm, and the distance between the centers of site 3 and site 4 is 5.0 cm. They are designed based on the average sizes of 13 adults. (C) Schematic diagram of Top PI layer with solder pad window.

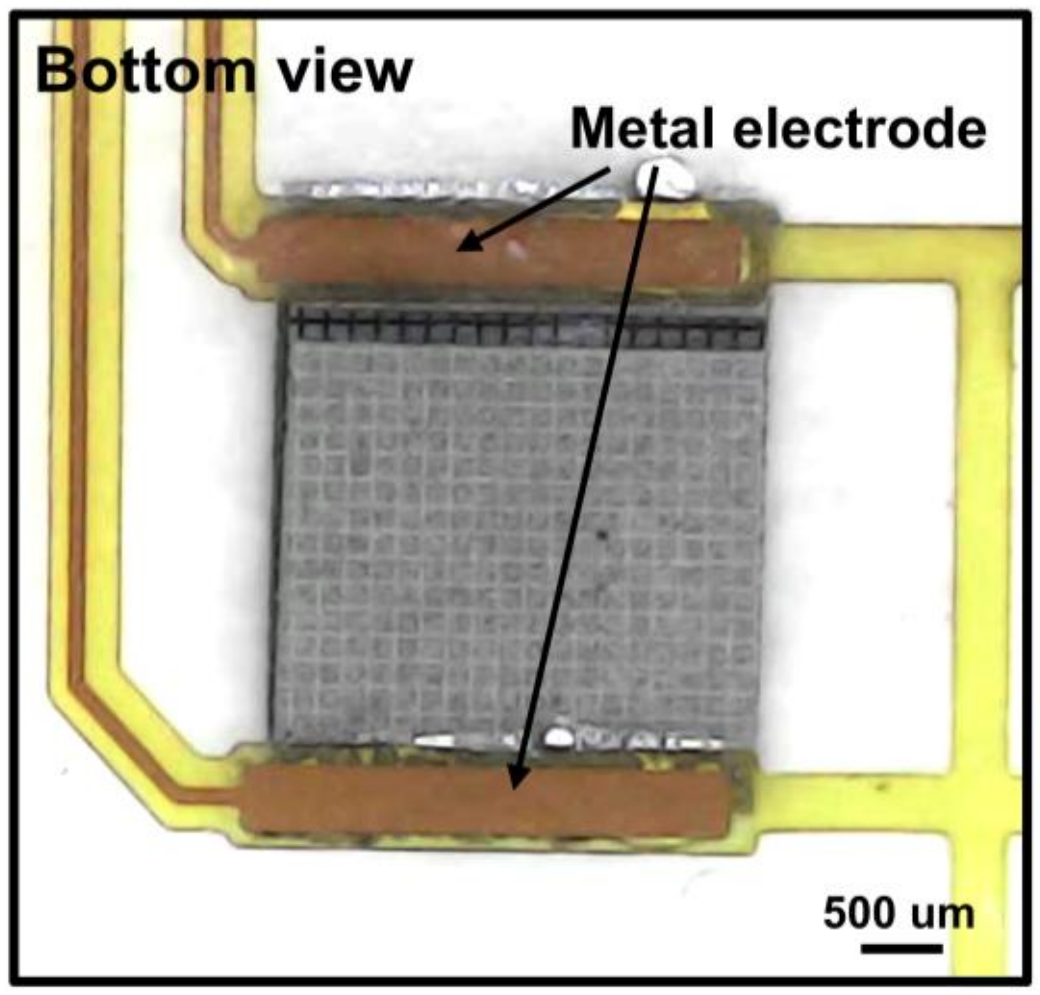

Fig. S2. Bottom view of soldering region of piezoelectric module.

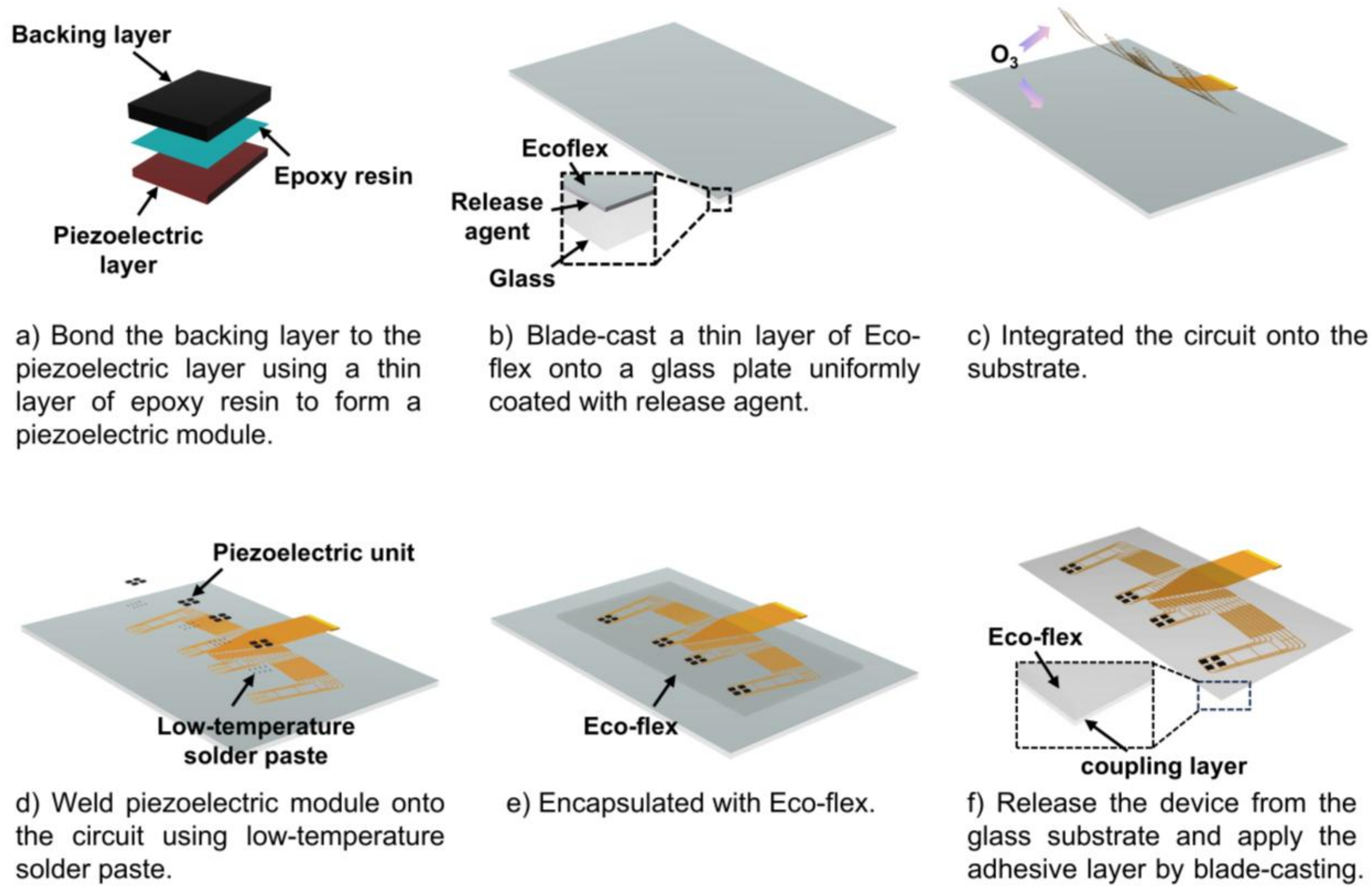

Fig. S3. Schematic fabrication process of stretchable ultrasound array.

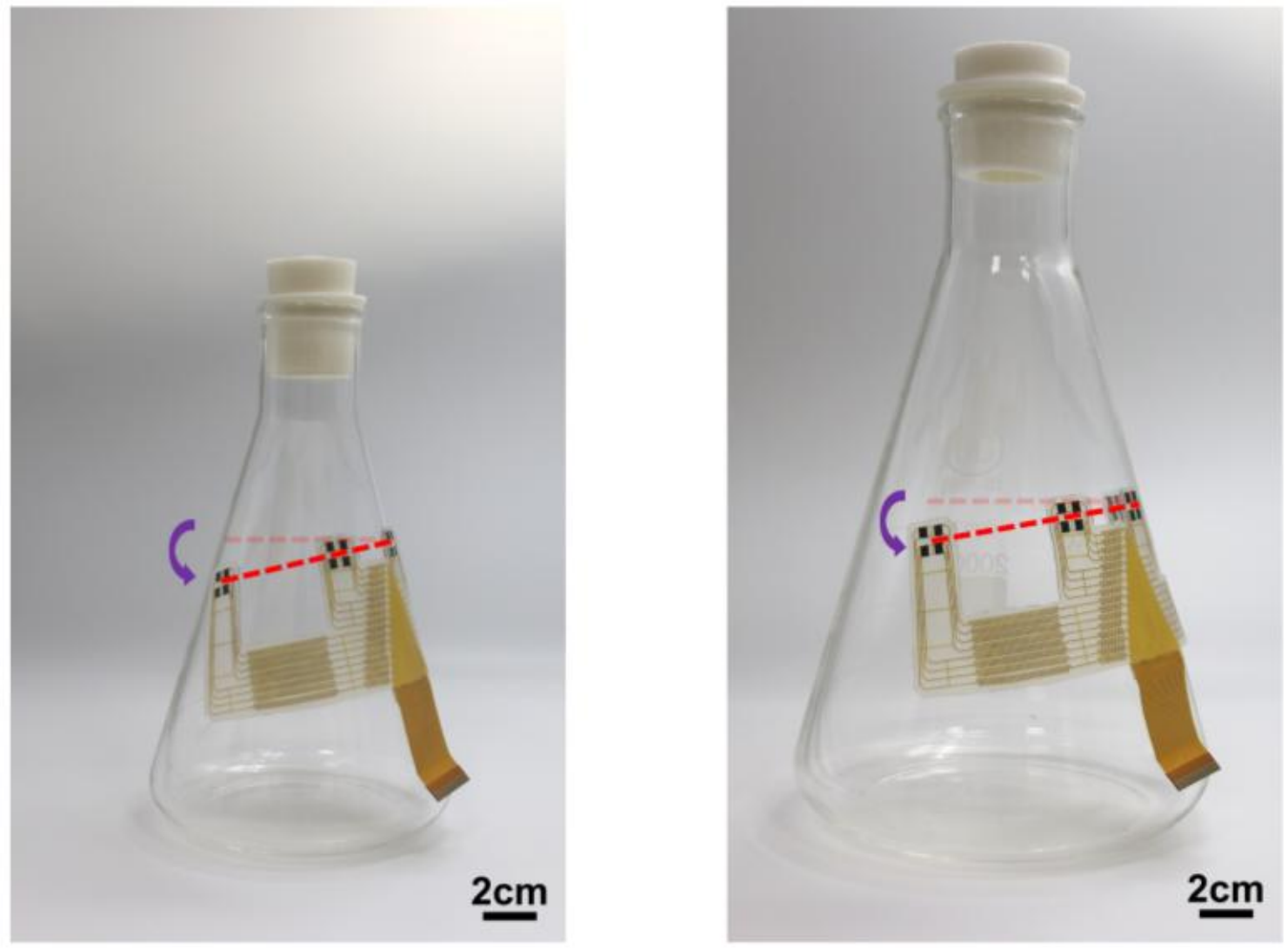

Fig. S4. Adhesion of stretchable multichannel ultrasonic device to conical flasks of varying curvatures without stretching. In the unstretched state, a geometric mismatch exists between the device and the lateral surfaces of conical flasks of different curvatures. This mismatch prevents the four functional sites from aligning horizontally, thereby impeding precise attachment to the target locations, which are required to lie along the same horizontal plane on the mid-forearm region.

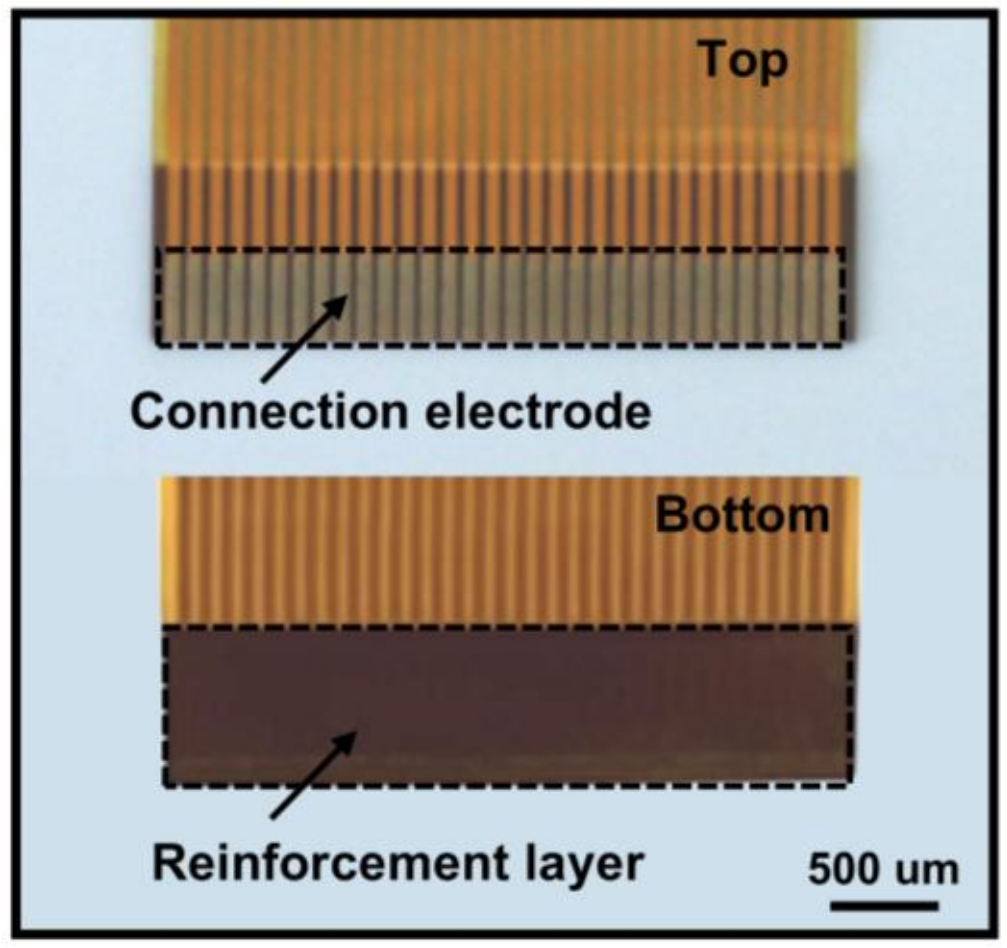

Fig. S5. Top and bottom view of the contact pad.

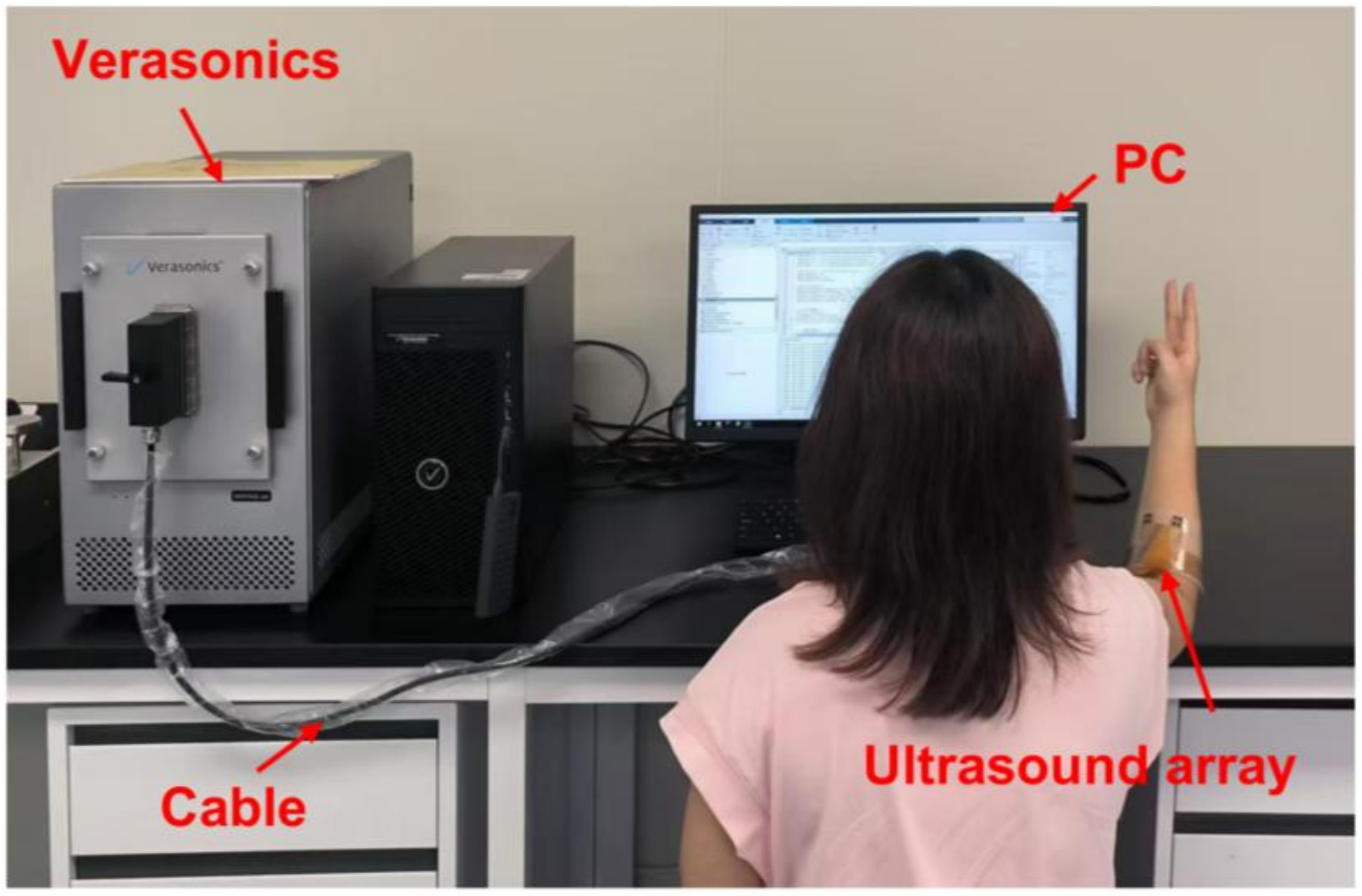

Fig. S6. Ultrasonic signal acquisition platform.

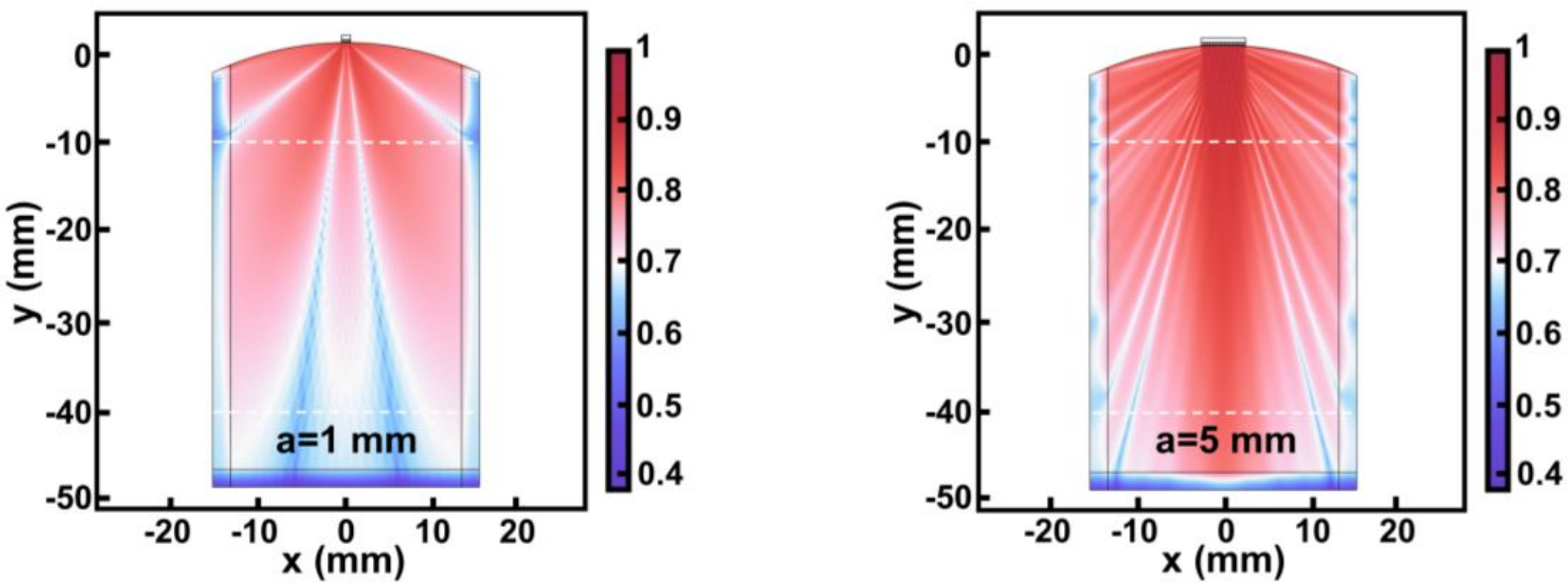

Fig. S7. Simulated acoustic fields with the piezoelectric module width of 1 mm (left) and 5 mm (right).

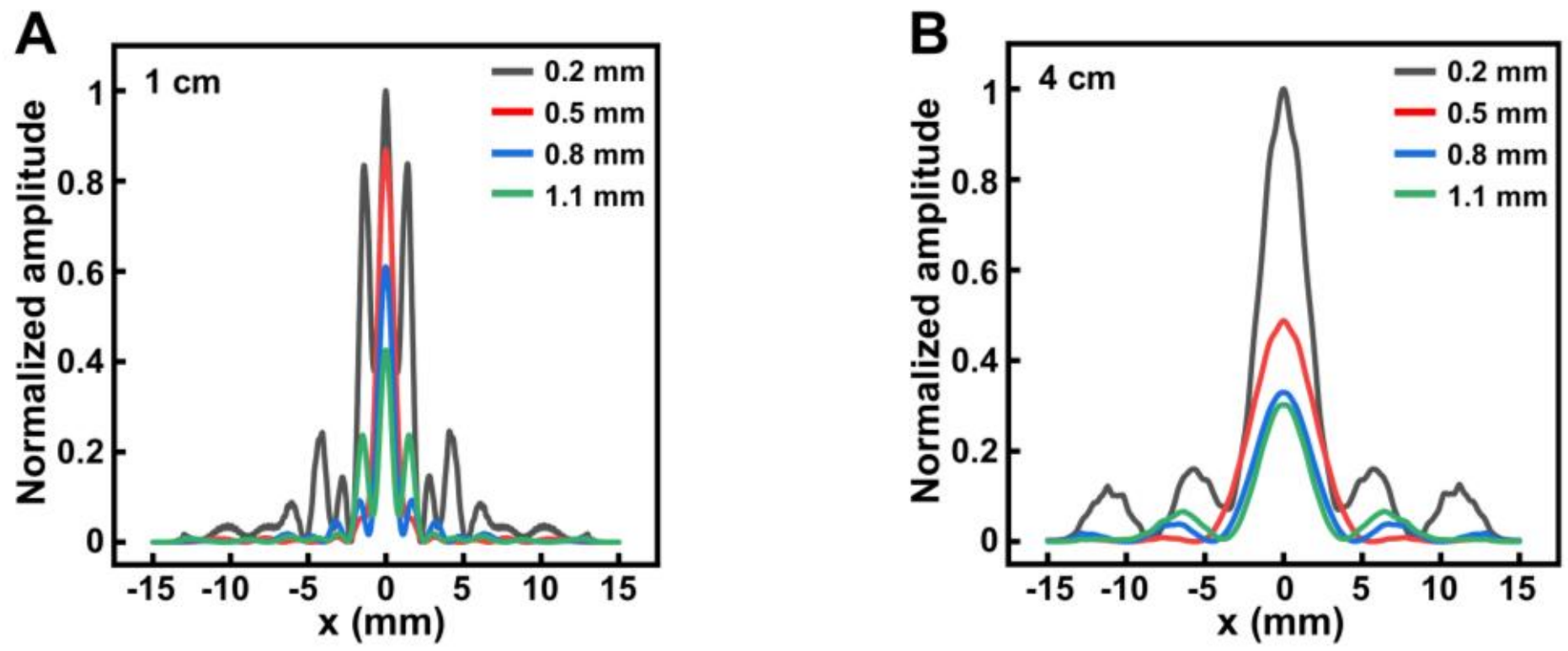

Fig. S8. Acoustic field profiles of the piezoelectric module of different height of backing layer (A) at the depth of 1 cm and (B) at the depth of 4 cm. To ensure the thin and lightweight characteristics of the device, we studied backing layer thicknesses within 1.1 mm and simulated different thicknesses (0.2 mm, 0.5 mm, 0.8 mm, 1.1 mm) respectively. It can be seen that the 0.5 mm backing layer maintains a relatively high acoustic intensity while exhibiting the best beam directivity. Therefore, we selected a 0.5 mm-thick backing layer for the experiment.

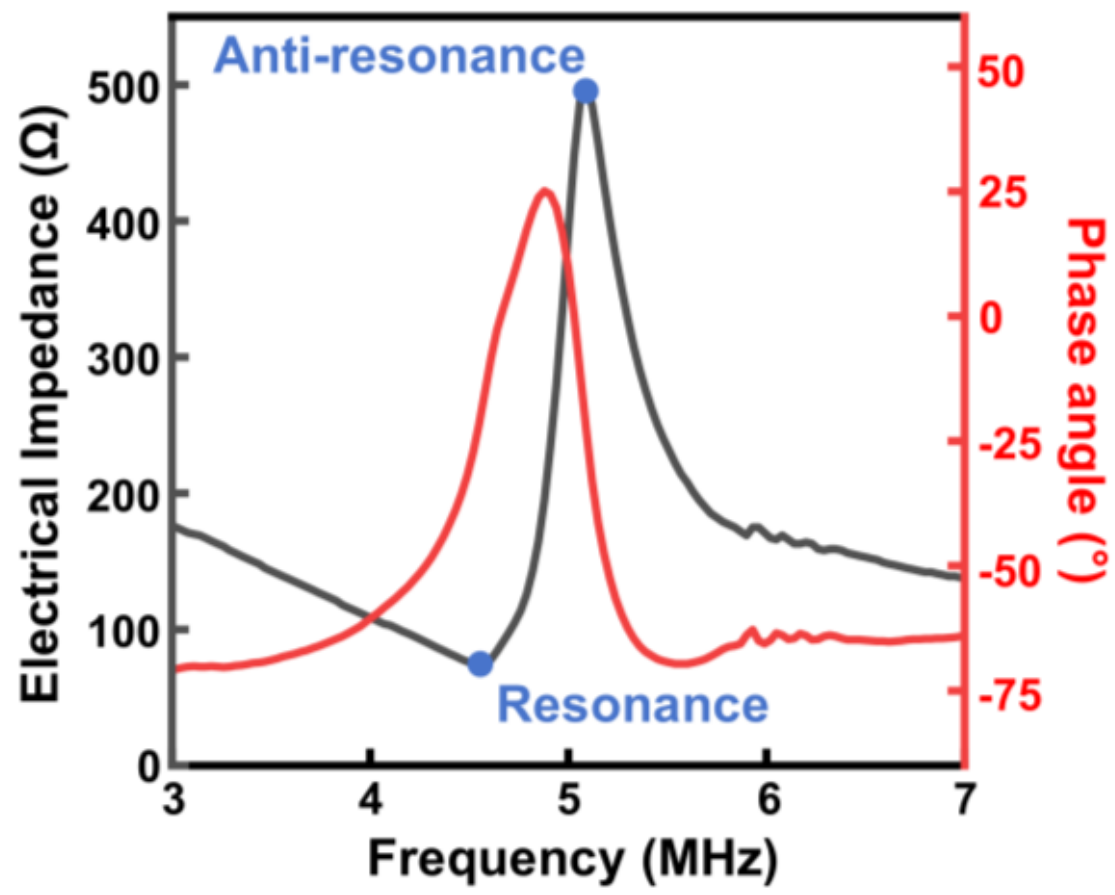

Fig. S9. Impedance and phase angle spectra of the 1-3 composite. The resonant and anti-resonant frequencies are labelled with solid circles.

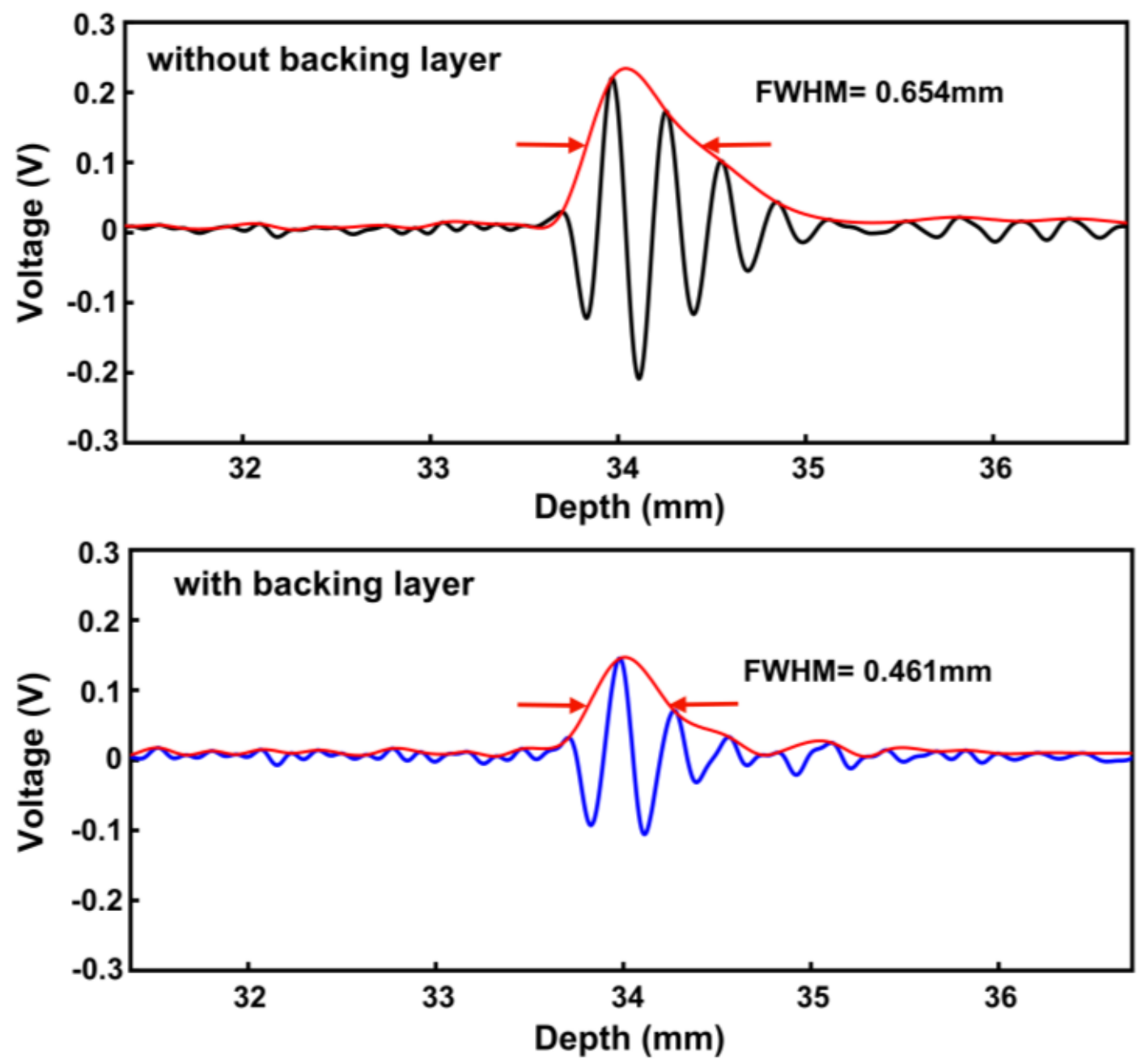

Fig. S10. Characterization of the axial resolution. Because the stretchable multichannel ultrasonic device operates at the A-mode, the axial resolution represents the spatial resolution. The full width at half maximum (FWHM) was calculated from the signal envelope to be 0.654mm and 0.461mm with(upper) and without(lower) backing layer. Therefore, the axial resolution is 0.654mm and 0.461mm, respectively. It indicates that the introduction of the backing layer has increased the longitudinal resolution by 29.5%.

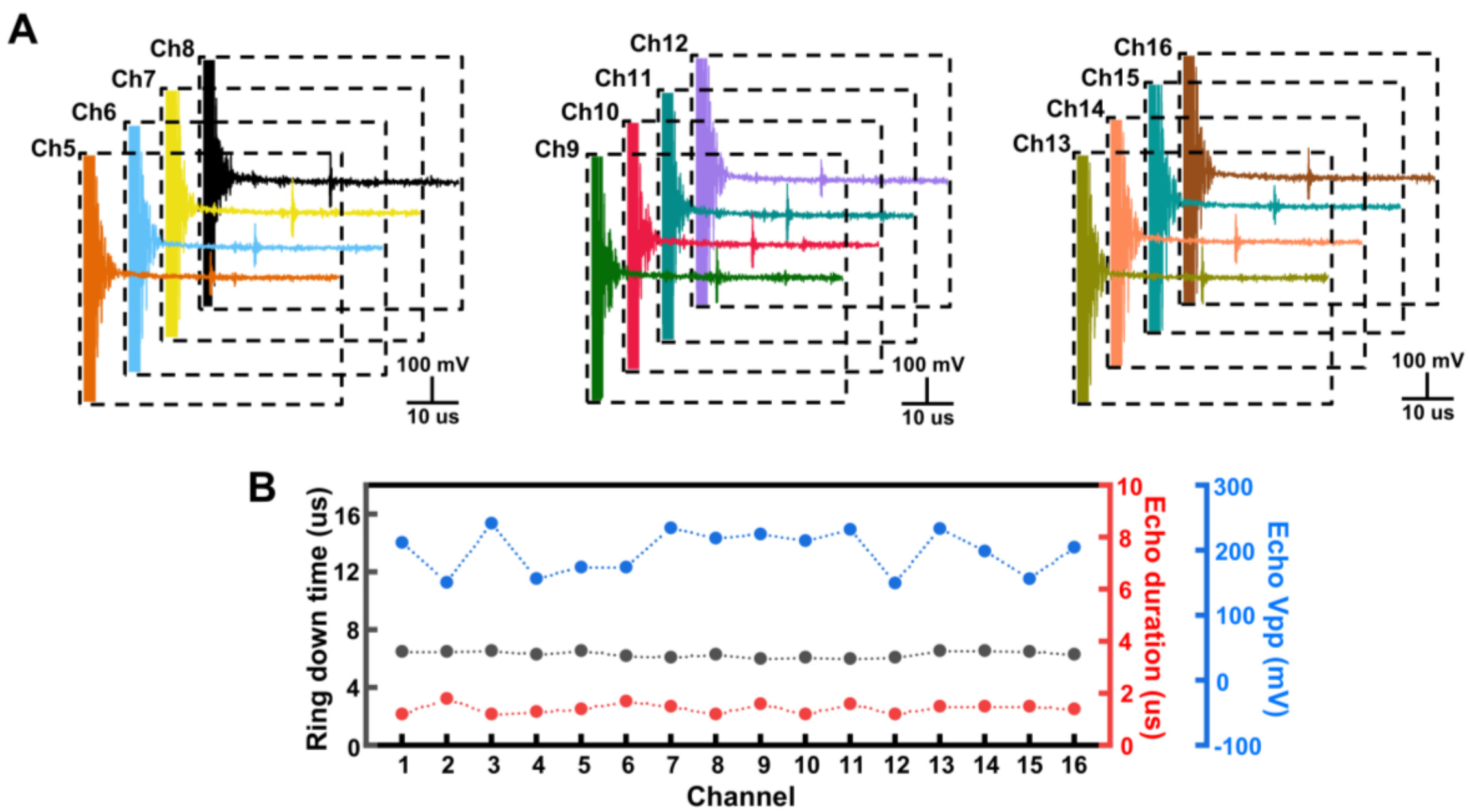

Fig. S11. Inter-channel consistency of the device. (A) The received signals of different channels and (B) the scatter plots of ring-down time, echo duration and V_{pp} value of the echo.

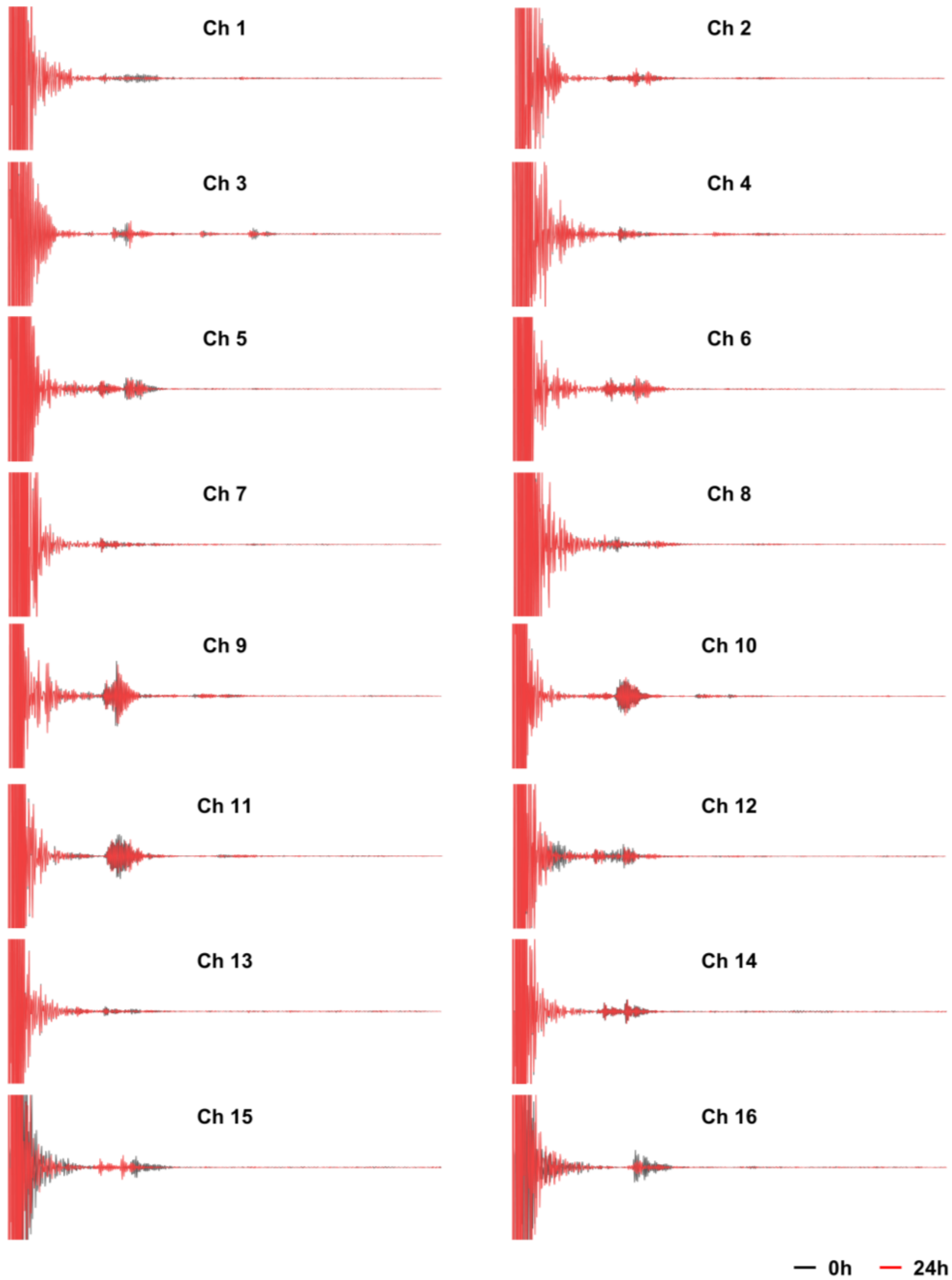

Fig. S12. The echo signal of 16 channels before and after 24-hour continuous usage when performing the "yes" gesture. After long-term continuous use, each channel of the device still maintains satisfactory transceiver performance, with no obvious degradation or distortion in the characteristic waveform and morphology of the echo signals, and good consistency in signal characteristics. Only slight variations occur in signal amplitude and time of flight, which mainly result from minor deformations, changes in fitting state, and local compression between the skin and the device during prolonged wearing, representing predictable and acceptable normal fluctuations.

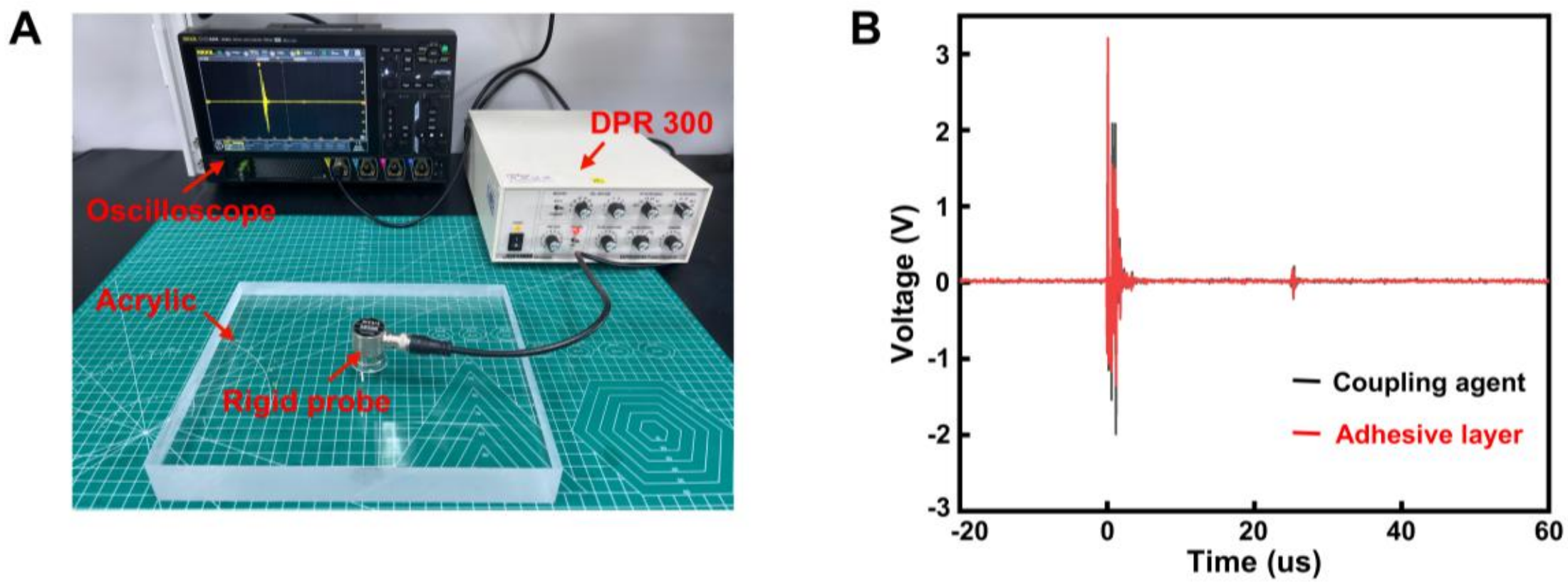

Fig. S13. Performance comparison between ultrasound coupling agent and adhesive layer. (A) Test plate. (B) The flexible adhesive layer performed equivalently to the ultrasound coupling agent with no discernible differences observed in signal reception by the rigid probe.

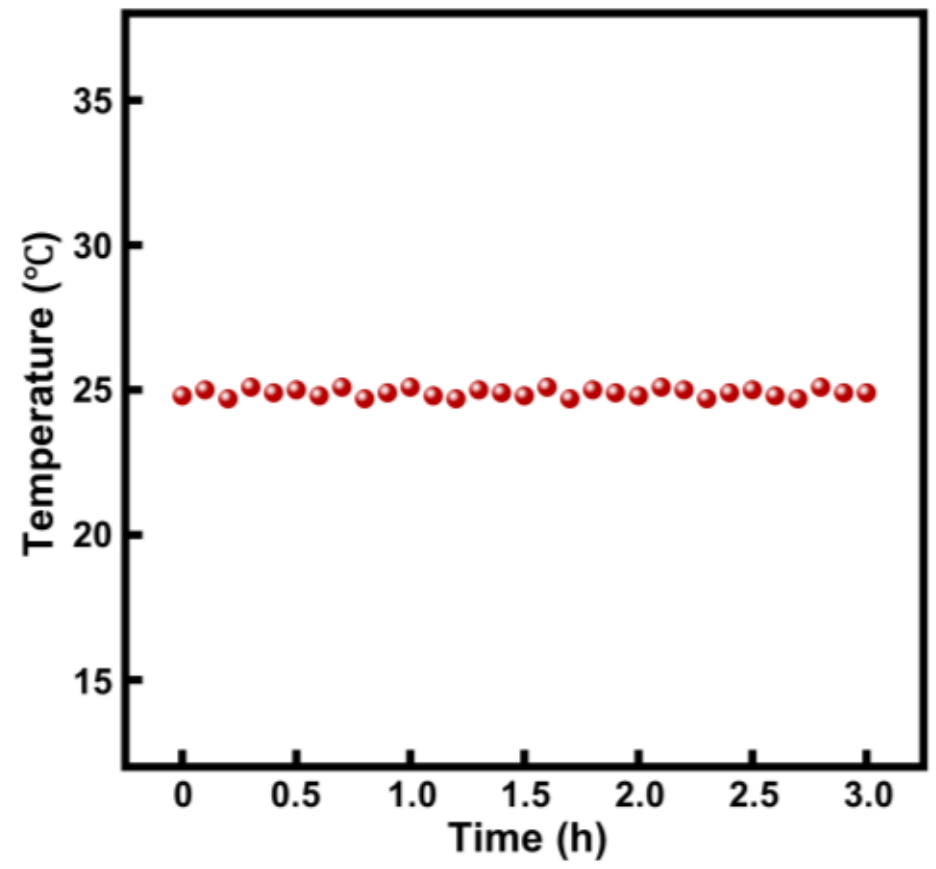

Fig. S14. Scatter plots of surface temperature variation of the device during continuous operation. The surface temperature of the device has always been maintained at room temperature (24.9 ± 0.2°C), indicating that the device will not generate heat during continuous operation and there is no risk of thermal damage.

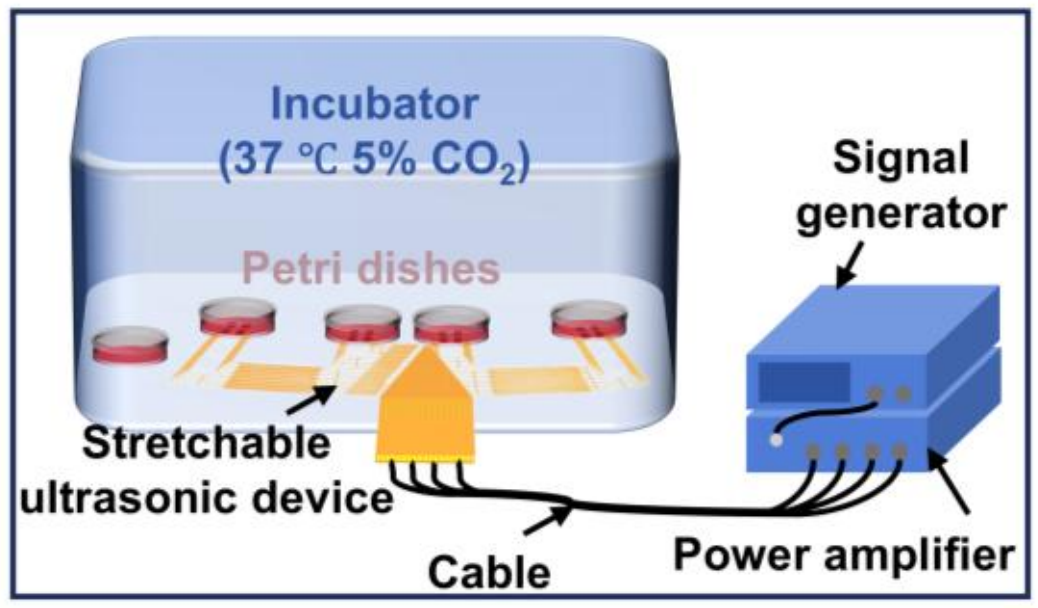

Fig. S15. Schematic diagram of the biocompatibility experiment setup.

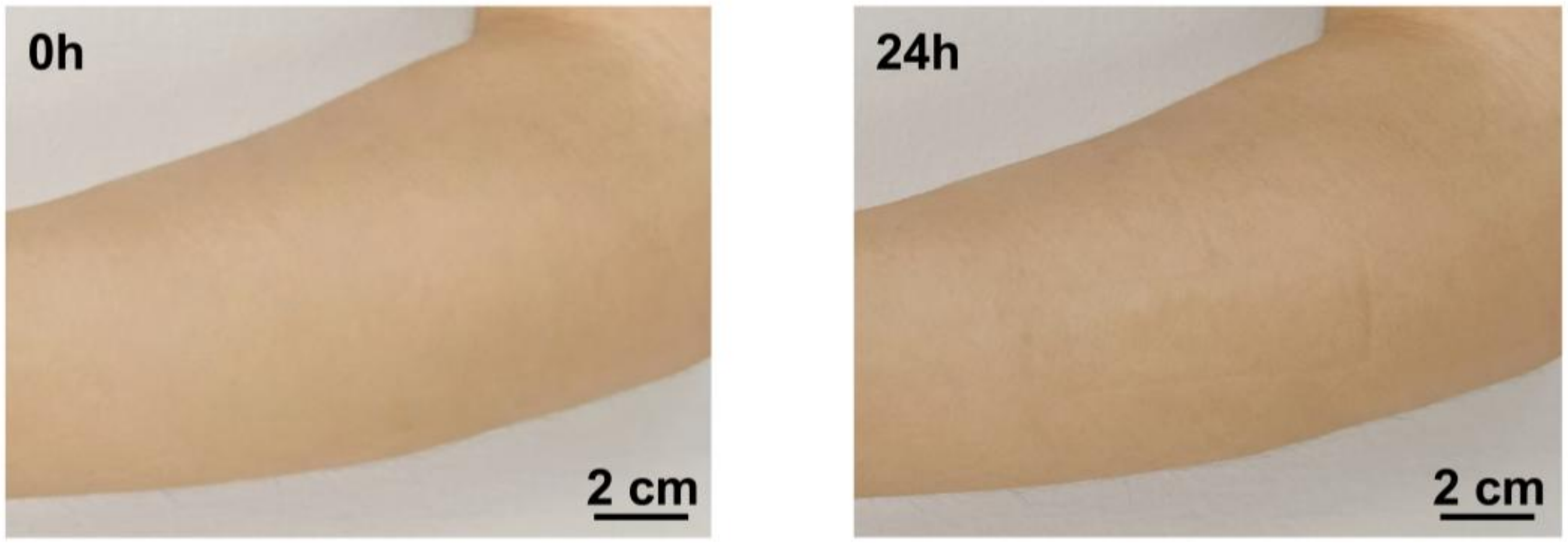

Fig. S16. 0 hour (left) and 24 hours (right) after the device is applied to the skin.

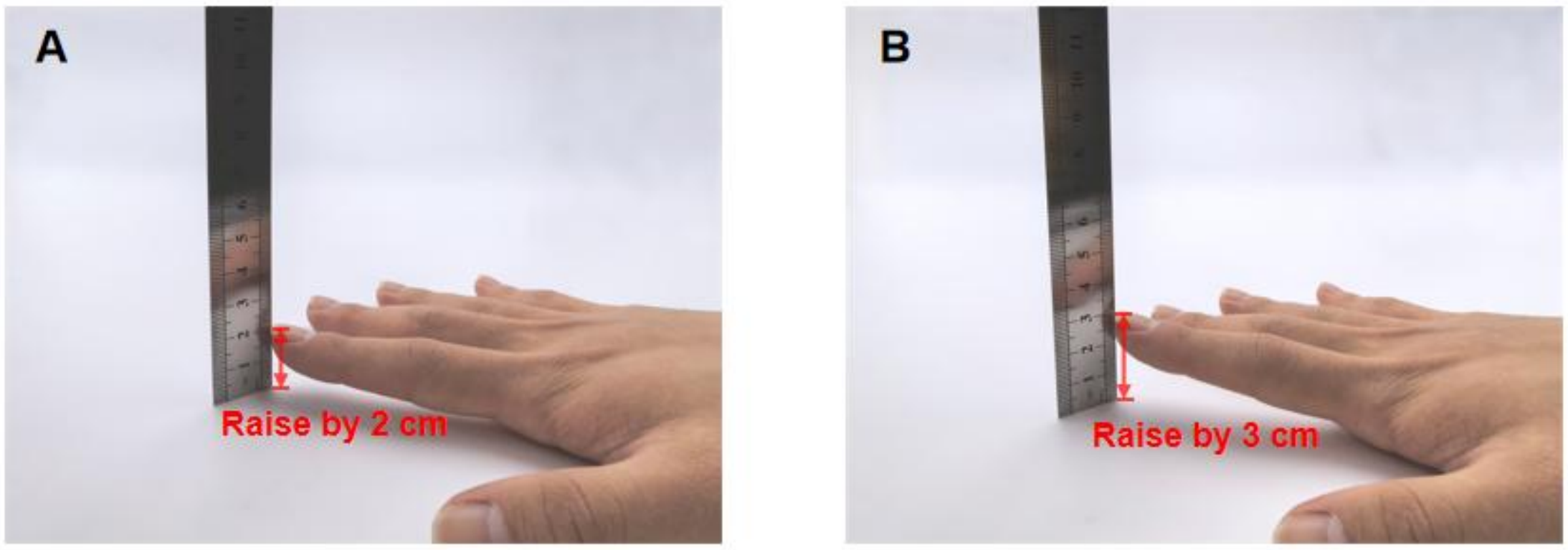

Fig. S17. Gesture calibration method. Gesture calibration is completed by aligning the fingertips with the ruler scales.

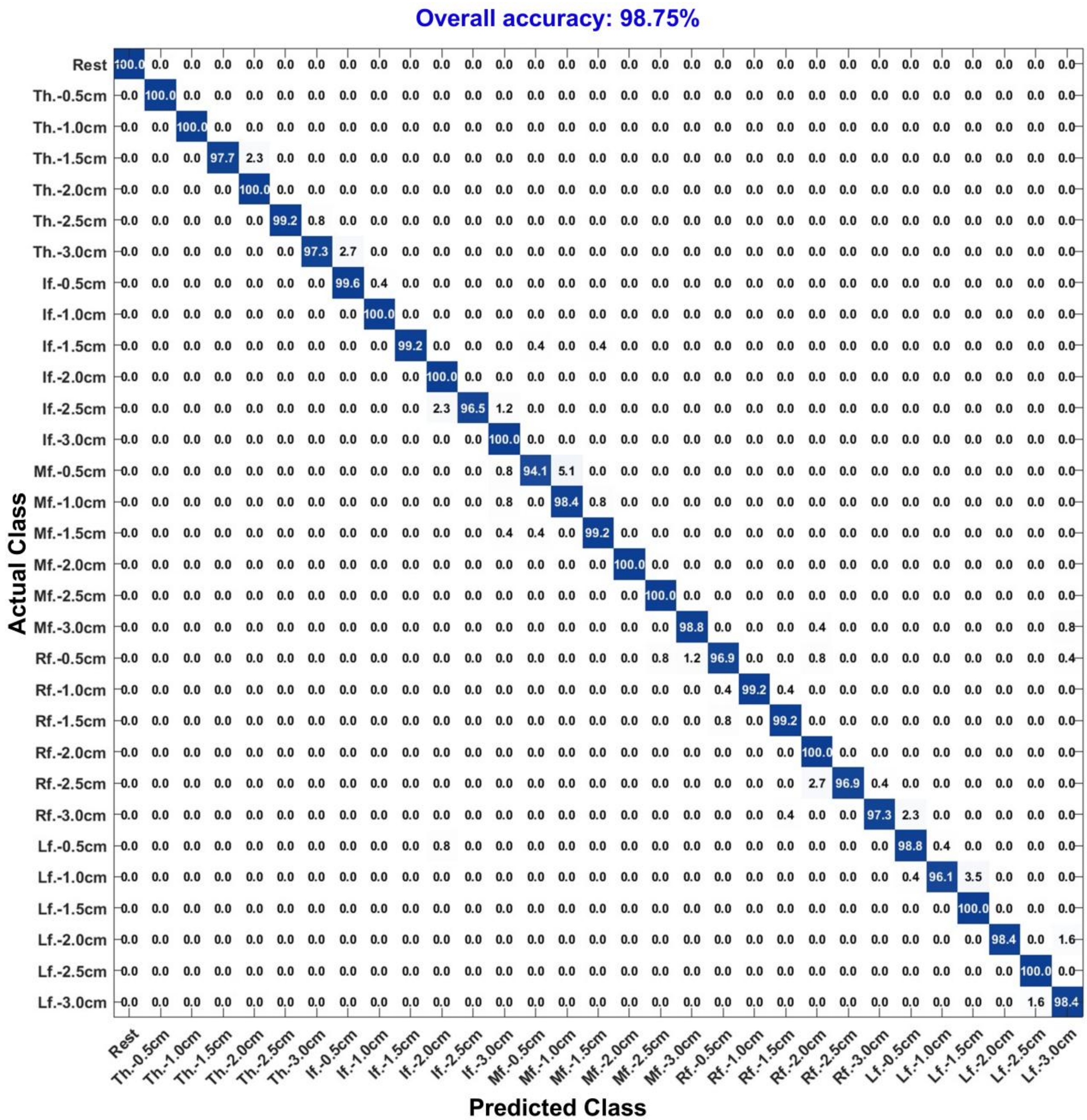

Fig. S18. Confusion matrix of the recognition accuracy.

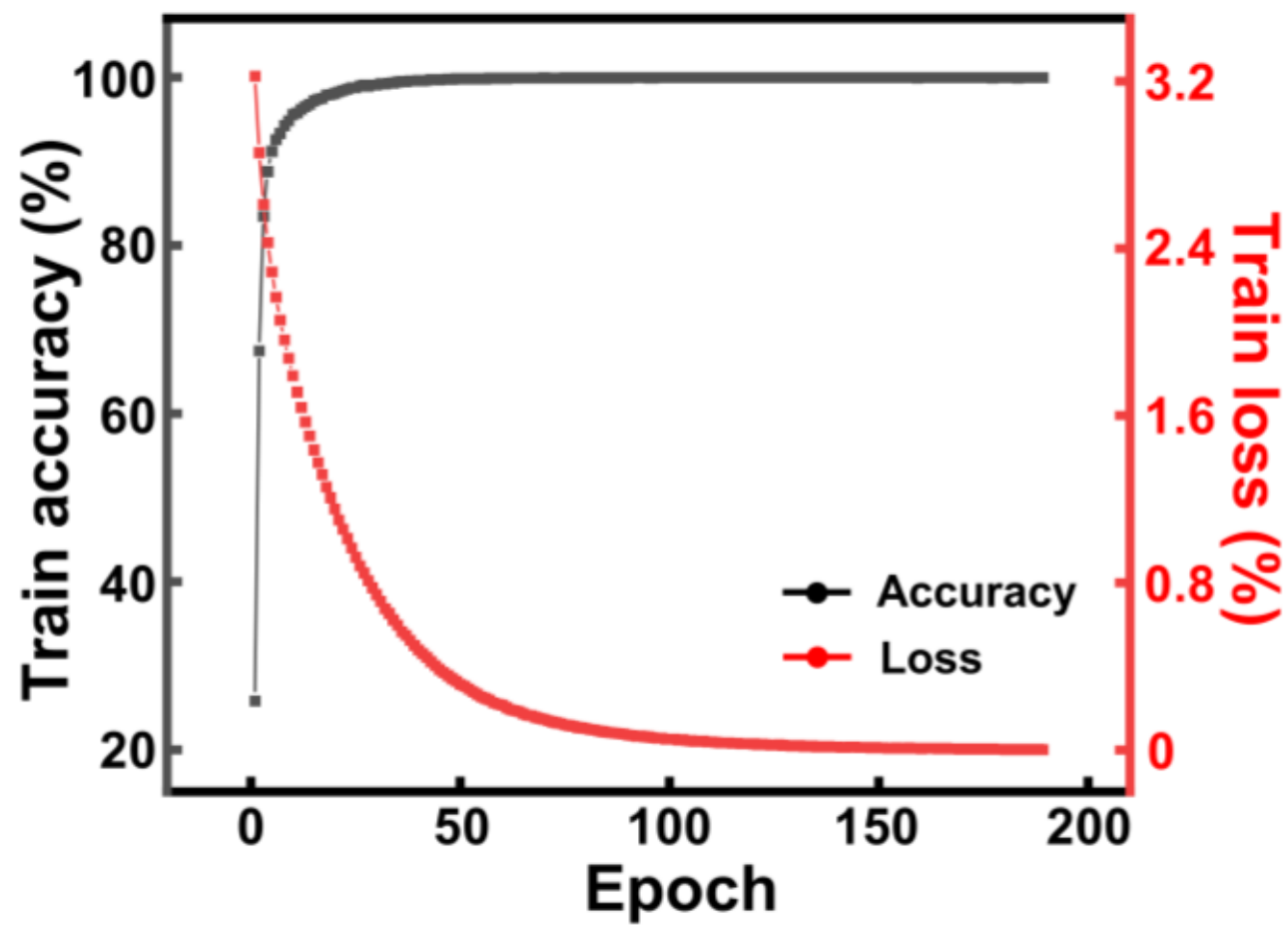

Fig. S19. The accuracy (black curve) and the loss (red curve) of the deep learning model during the training progress.

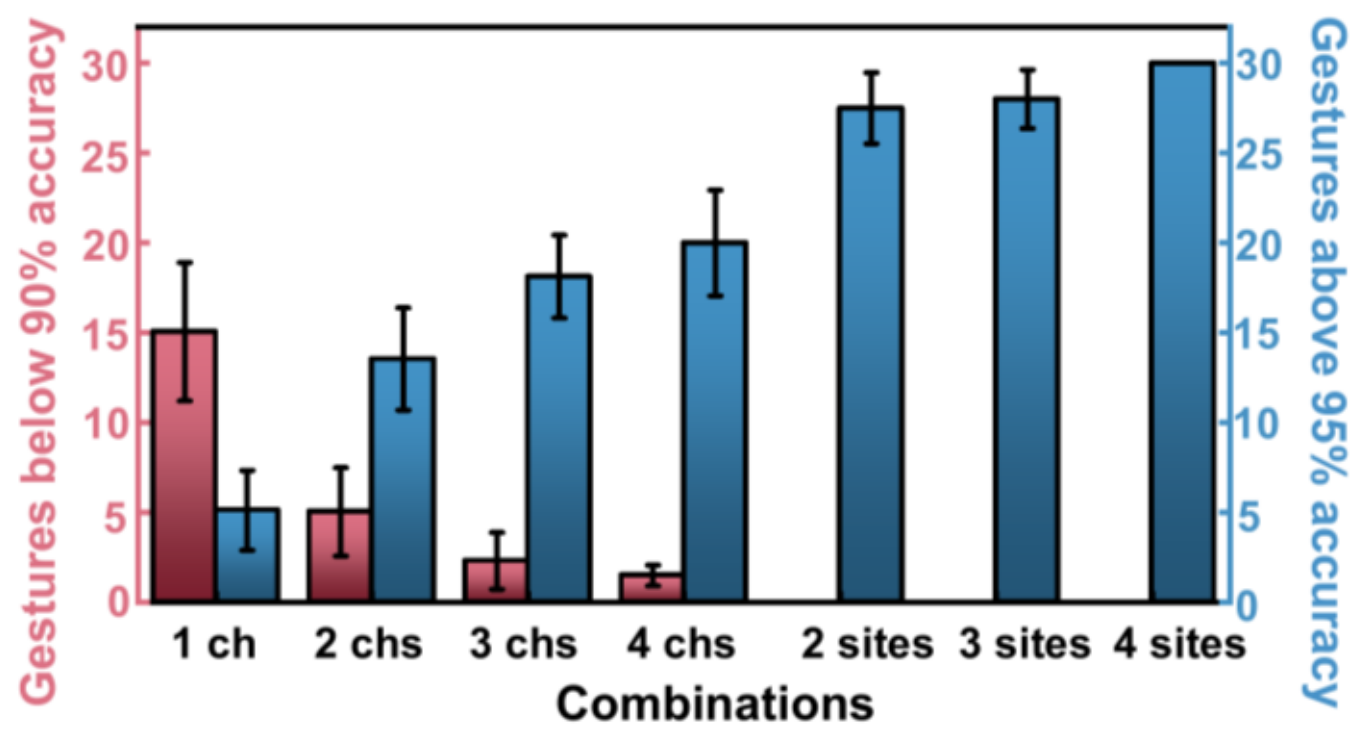

Fig. S20. Effect of channel combinations on the distribution of recognition accuracy across gestures. Data are presented as mean ± SD. The 1 ch, 2 chs, 3 chs, 4 chs, 2 sites, 3 sites, 4 sites results were calculated across 16 single-channel selections, 24 two-channels selections, 16 three-channels selections, 4 four-channels selections, 6 two-sites selections, 4 three-sites selections and 1 four-sites selection respectively. The selections were treated as technical replicates.

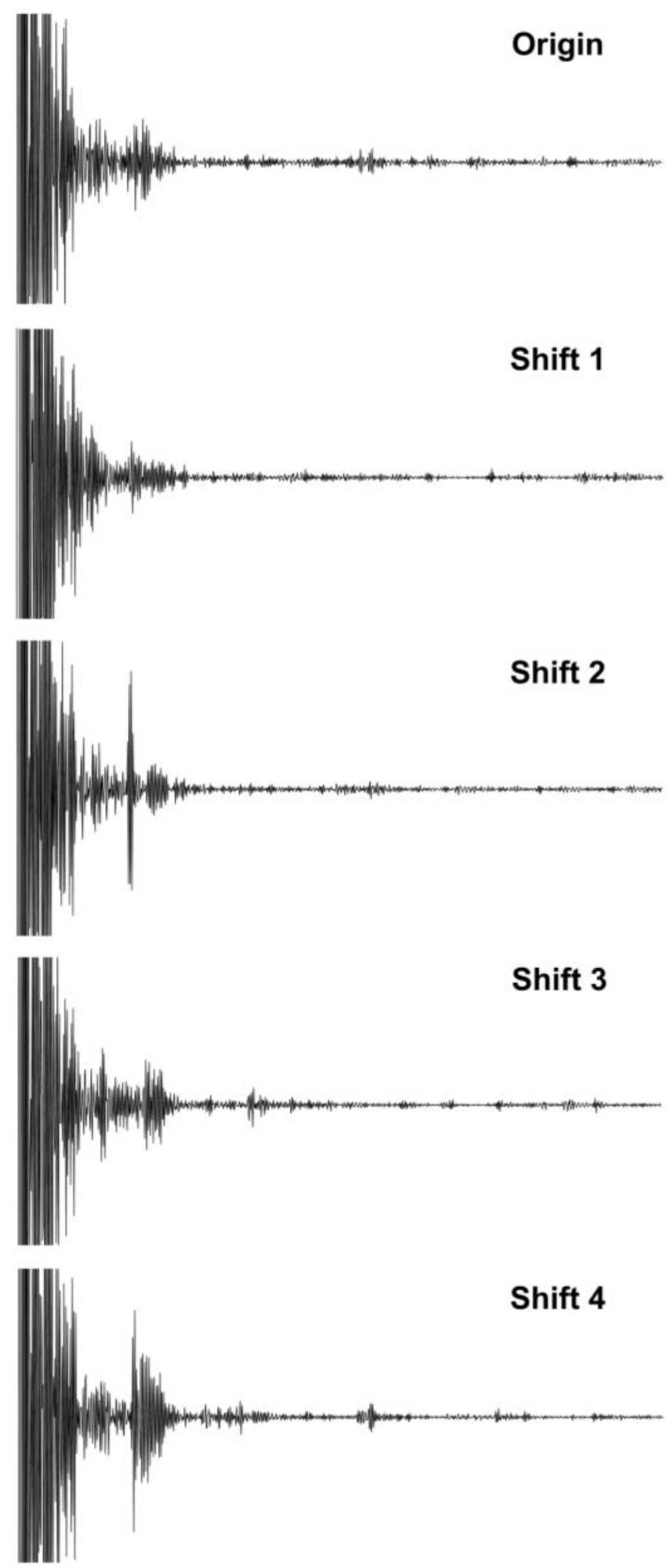

Fig. S21. Single-channel ultrasound signal waveform at different attachment positions when the index finger is lifted by 3 cm.

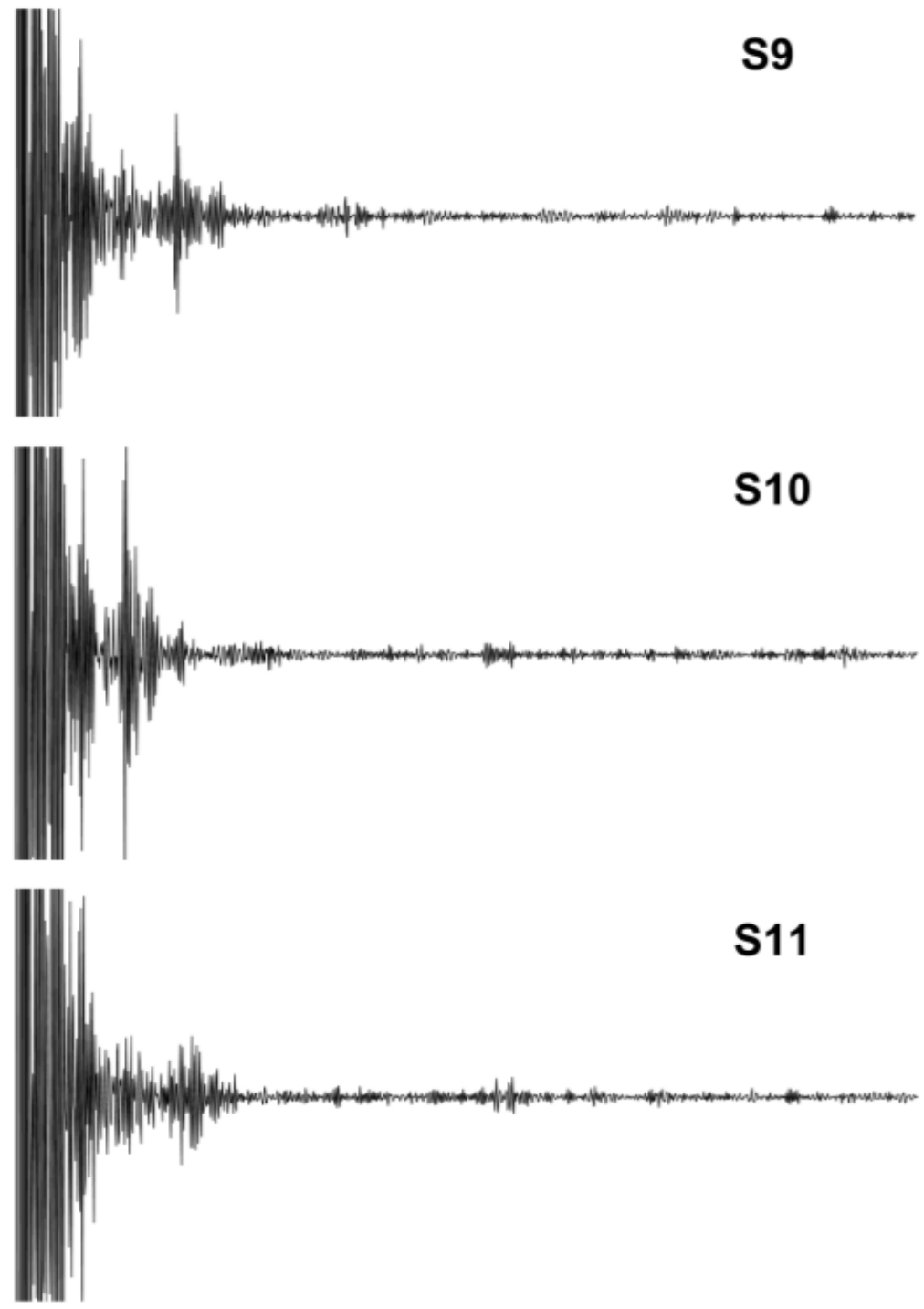

Fig. S22. Single-channel ultrasound signal waveform across different users when the index finger is lifted by 3 cm.

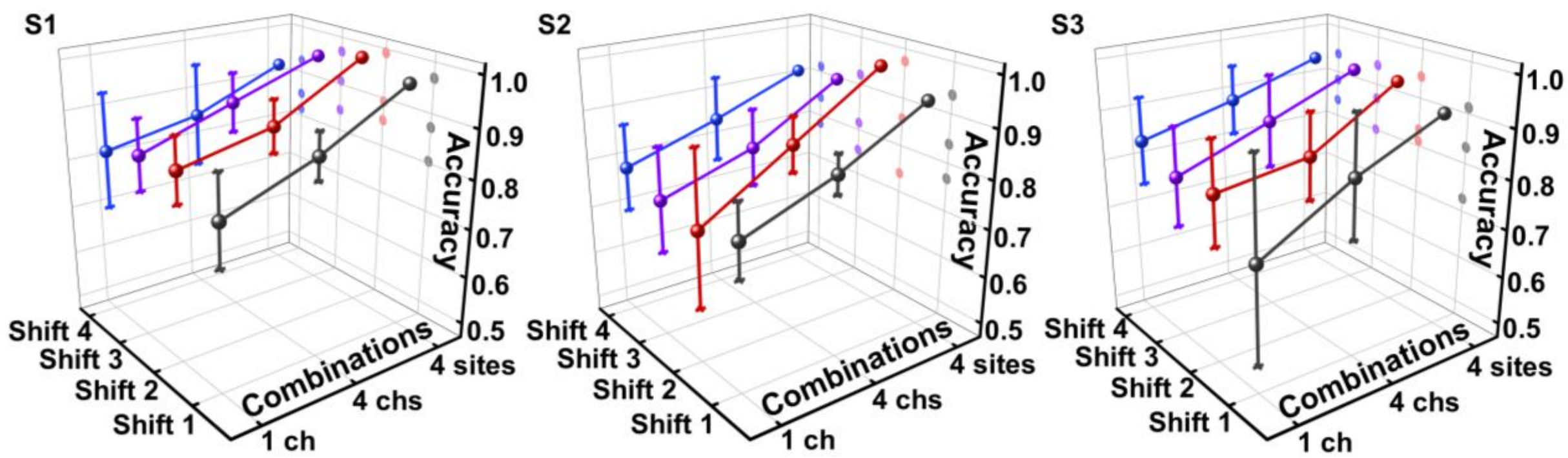

Fig. S23. Individual-subject recognition accuracy of different channel configurations under shifted positions after lightweight calibration, mean ± SD, technical replicates.

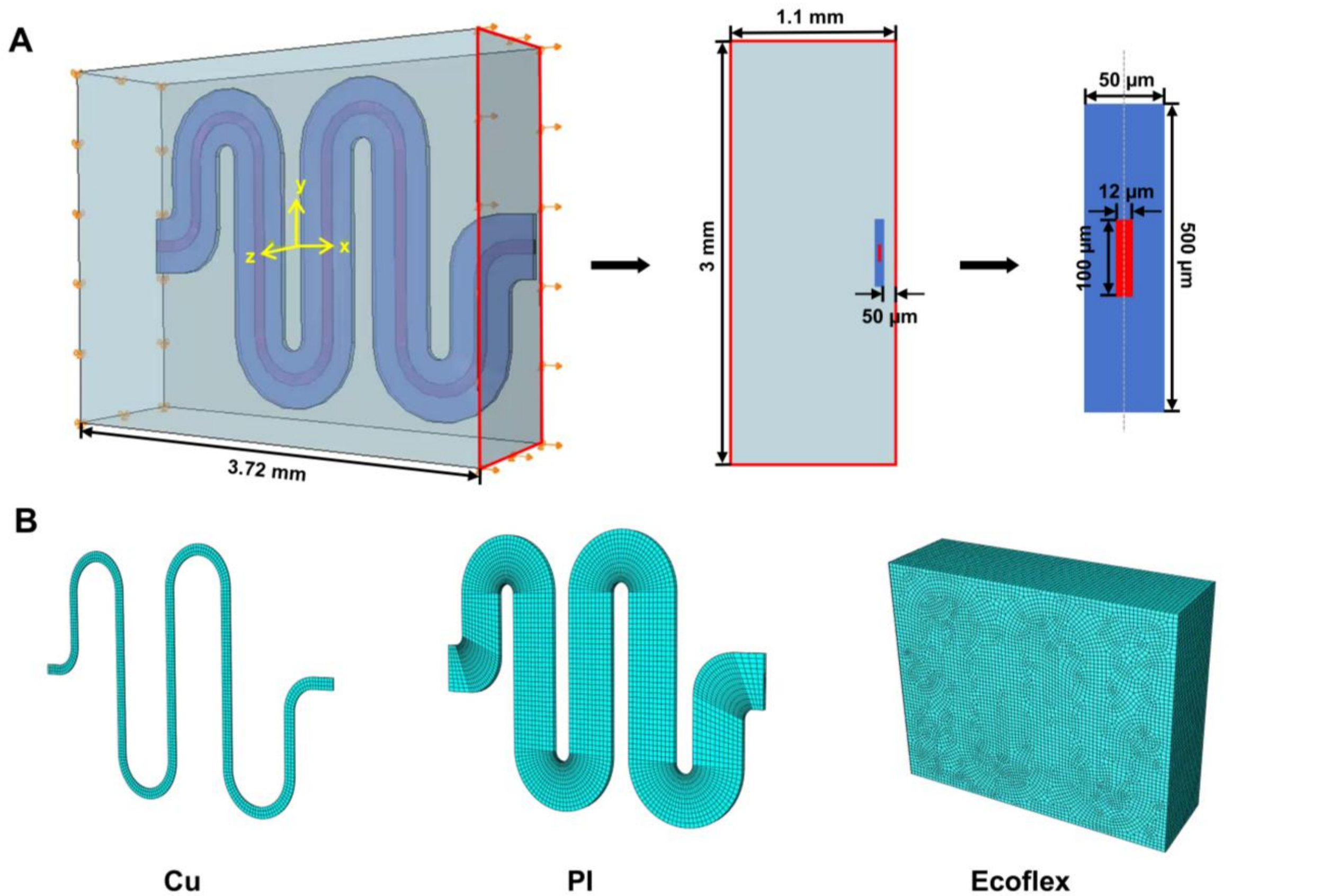

Fig. S24. Finite element model for the deformation of serpentine wire. (A) Structural dimensions and boundary conditions, the left end face is restricted with U_x=U_y=0, while the right end face is subjected to displacement U_x=1.12mm. (B) Meshing of components.

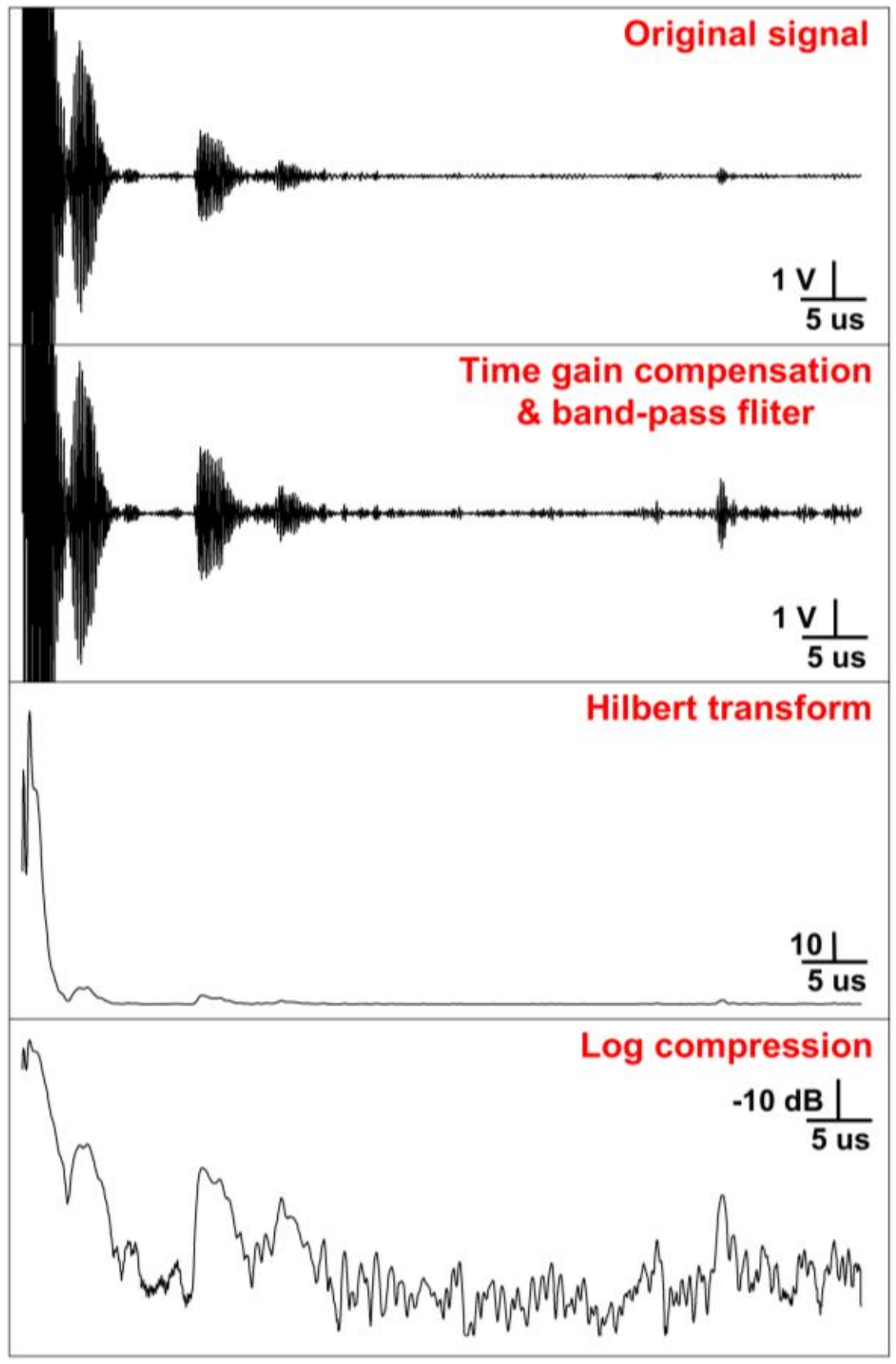

Fig. S25. Signal preprocessing process.

Component	Material	Source
encapsulation layer	Ecoflex 00-30	Smooth-On, USA
backing layer	Composite of epoxy resin, alumina powder, and mica flakes	Hunan Xiongwu Technology Co., Ltd., China
epoxy adhesive layer	Epo-tek 301	Epoxy Technology, 438 USA
piezoelectric layer	1-3 piezoelectric composite material	Baoding Xinwei Electronic Technology Co., Ltd., China
welding agent	low-temperature solder paste KZ-1513	KELLYSHUN, China
circuit layer	FPC	Shenzhen Hongyang Circuit Technology Co., Ltd., China
substrate layer	Ecoflex 00-30	Smooth-On, USA
pressure-sensitive adhesive layer	Derma-Tac silicone-based pressure-sensitive adhesive	Smooth-On, USA

Table S1. Summary of material information for stretchable multi-channel ultrasonic devices.

Material \ Parameters	Density (kg/m^3)	Acoustic velocity (m/s)	Young's Modulus (GPa)	Poisson's Ratio	Attenuation coefficient (dB/m)
PZT-5H	7500	—	—	—	—
Epoxy	1673	—	1	0.38	—
Eco-flex	1070	972	—	—	1987.36
Tissue	1044	1568	—	—	500
Backing layer	1754.38	—	6.32	0.37	—

Table S2. Material constants for COMSOL.

Subject ID	Gender	Age	Ethnicity
S1	Male	25	Han
S2	Male	23	Han
S3	Male	28	Han
S4	Male	23	Han
S5	Male	26	Han
S6	Female	25	Han
S7	Female	22	Han
S8	Female	25	Han
S9	Male	33	Han
S10	Male	29	Han
S11	Female	26	Han

Table S3. Detailed information of 8 healthy subjects (S1-S8) for model training and 3 new subjects (S9-S11) for test.

Transducer type	Sensing mode	Channel number	Wearable circuit	Classification use cases	Kinds of motions	Accuracy	Reference
sEMG device	sEMG	6	No	Coarse discrete daily gestures	15	98%	*(49)*
		57	No		7	~98%	*(50)*
		8	Yes		18	97%	*(13)*
		59/70/75	Yes		20	97.3%	*(51)*
		64	Yes		21	92.87%	*(11)*
		16/12	Yes	Ninapro DB5/DB7	41	93.87% / 91.6%	*(52)*
		6	Yes	ASL alphabet	26	93.6%	*(53)*
Ultrasound	B-mode	N/A	No	Coarse discrete daily gestures	15	91%	*(20)*
		N/A	No		6	94%	*(54)*
	A-mode	4	No		6	96.5%	*(44)*
		2	No		12	96%	*(55)*
		4	No		12	98.83%	*(39)*
		4	No		10	95.26%	*(56)*
		9	No		10	96%	*(57)*
		1	No		8	67.01%	*(58)*
		16	**Yes**	**Fine-grained finger movements (MCP joint variation < 5°)**	31	**98.75%**	**This work**

Table S4. Comparison of discrete gesture recognition based on sEMG and ultrasound.

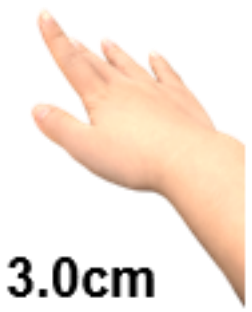

If. 3.0cm

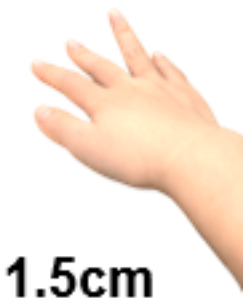

Rf. 1.5cm

**Combination**		**Accuracy for If. 3.0cm**	**Accuracy for Rf. 1.5cm**
1Ch	Ch9	87.90%	88.30%
	Ch10	90.60%	78.90%
	Ch11	92.20%	81.20%
	Ch12	78.50%	78.90%
	Avg.	87.30%	81.83%
2Chs	Ch9&Ch10	88.30%	96.50%
	Ch9&Ch11	90.20%	89.10%
	Ch9&Ch12	88.30%	84.80%
	Ch10&Ch11	86.70%	87.50%
	Ch10&Ch12	85.90%	93.40%
	Ch11&Ch12	86.30%	96.00%
	Avg.	87.60%	91.20%
3Chs	Ch9&Ch10&Ch11	96.50%	97.30%
	Ch9&Ch10&Ch12	93.80%	95.70%
	Ch9&Ch11&Ch12	87.90%	91.80%
	Ch10&Ch11&Ch12	87.50%	93.00%
	Avg.	91.43%	94.45%
4Chs	Ch9 to Ch12	92.60%	96.90%

Table S5. The recognition of specific gestures by different channel combinations within a single site. Analyze the specific gestures. Take raising the index finger by 3.0cm and the ring finger by 1.5cm as examples. Within a single functional site 2, as the number of channels increased, the recognition accuracy of both showed an upward trend. When all four channels were used, the recognition accuracy reached 92.60% and 96.90%, respectively. From the data in the table, it can be found that if a certain channel combination is used alone, it is almost impossible to achieve the highest recognition accuracy for two gestures simultaneously, and this is even more such situation when extended to 31 gestures. Therefore, only when all four channels within a single site are used can the best performance indicators of balanced high precision that the current single-site design can achieve be reached for all 31 gestures.

	Combination	Accuracy for If. 3.0cm	Accuracy for Rf. 1.5cm
1 site	Ch1 to Ch4	99.20%	96.90%
	Ch5 to Ch8	92.20%	96.90%
	Ch9 to Ch12	92.60%	96.90%
	Ch13 to Ch16	94.10%	85.90%
	Avg.	94.53%	94.15%
2 sites	Ch9 to Ch12 & Ch1 to Ch4	97.70%	99.60%
	Ch9 to Ch12 & Ch5 to Ch8	98.80%	96.10%
	Ch9 to Ch12 & Ch13 to Ch16	98.40%	98.00%
	Avg.	98.30%	97.90%
3 sites	Ch9 to Ch12 & Ch1 to Ch4 & Ch5 to Ch8	98.40%	98.80%
	Ch9 to Ch12 & Ch1 to Ch4 & Ch13 to Ch16	98.80%	98.00%
	Ch9 to Ch12 & Ch5 to Ch8 & Ch13 to Ch16	98.00%	98.40%
	Avg.	98.40%	98.40%
4 sites	16 Channels	100.00%	99.20%

Table S6. The recognition of specific gestures by different combinations of functional sites. Similarly, for the combination of different functional sites, as the number of participating functional sites increases, the recognition accuracy of both gestures shows an upward trend. When all four functional sites are used, the recognition accuracies reach 100% and 99.20%, respectively, achieving extremely high recognition precision. If a single functional site combination is used alone, it is almost impossible to achieve the highest recognition accuracy for two gestures simultaneously, and this is even more such situation when extending to 31 gestures. Therefore, only when all four functional sites are used can the best performance indicators of balanced and high-precision achievable by the current device design be reached for all 31 gestures.

Strategy	Channel combination	S1 (%)	S2 (%)	S3 (%)	S4 (%)	S5 (%)	S6 (%)	S7 (%)	S8 (%)
Space	[1, 5, 9, 13]	97.58	97.68	99.4	95.06	95.36	93.95	97.88	99.60
	[2, 6, 10, 14]	97.68	96.57	99.7	94.76	93.65	95.56	97.58	98.69
	[3, 7, 11, 15]	97.18	98.69	97.18	97.78	96.27	93.55	98.29	98.49
	[4, 8, 12, 16]	97.28	98.59	98.39	96.17	96.47	93.95	98.69	98.29
	Mean	**97.43**	**97.88**	**98.67**	**95.94**	**95.44**	**94.25**	**98.11**	**98.77**
	SD	**0.24**	**0.99**	**1.14**	**1.37**	**1.29**	**0.89**	**0.48**	**0.58**
Density	[1, 2, 3, 4]	98.49	96.07	97.38	91.83	96.88	95.16	93.25	95.77
	[5, 6, 7, 8]	96.88	95.16	98.39	98.49	94.15	93.04	97.48	95.87
	[9, 10, 11, 12]	95.46	96.47	93.75	96.17	96.47	94.66	97.98	98.89
	[13, 14, 15, 16]	93.45	96.27	97.58	96.67	96.17	91.13	96.98	96.98
	Mean	**96.07**	**95.99**	**96.78**	**95.79**	**95.92**	**93.5**	**96.42**	**96.88**
	SD	**2.14**	**0.58**	**2.06**	**2.82**	**1.21**	**1.82**	**2.15**	**1.45**

Table S7. Recognition accuracy of different four-channel selection strategies across subjects.

	Hyperparameter	Description
**Model structure**	Model architecture	Multi-Scale 1D CNN
	Classes number	31
	Channel number	16
	Multiscale kernels	[1, 3, 5, 7, 11]
	Residual connections	true
**Basic training**	Optimizer	Adam
	Learning rate	0.0001
	Batch size	64
	epochs	300
	Early stopping patience	8
	Loss function	Cross Entropy Loss
	Train/val/test split	70/15/15
**Adaptive calibration**	Model	Enhanced Calibration Model
	Optimizer	AdamW
	Learning rate	0.002
	Batch size	64
	Epochs	60
	Scheduler	Cosine Annealing Warm Restarts (T_0 =15, T_mult =2)
	Frozen backbone	partial (unfreeze Conv30 and Conv40)

Table S8. Model hyperparameters.

Group	Channels	Calibration (Yes or No)	Origin			Shift 1			Shift 2			Shift 3			Shift 4		
			S9(%)	S10(%)	S11(%)	S1(%)	S2(%)	S3(%)	S1(%)	S2(%)	S3(%)	S1(%)	S2(%)	S3(%)	S1(%)	S2(%)	S3(%)
1 ch	Ch 1	No	3.23	1.56	0.44	3.89	2.89	3.67	4.34	4.00	2.89	2.89	8.79	1.78	3.11	3.45	5.78
		Yes	90.66	93.99	96.66	88.43	80.42	93.44	98.33	87.88	98.78	92.44	91.77	83.09	95.66	87.88	97.44
	Ch 2	No	5.67	0.67	1.22	6.45	0.89	3.45	3.11	5.56	5.45	3.00	3.11	5.01	8.12	4.78	4.00
		Yes	96.22	95.11	97.22	96.00	86.54	97.22	97.11	93.33	96.44	95.44	91.43	93.99	93.33	91.10	92.10
	Ch 3	No	5.78	2.00	1.67	8.90	2.78	4.34	4.56	1.45	3.00	3.45	4.67	4.67	3.00	2.78	0.33
		Yes	96.00	96.00	92.55	93.88	77.42	92.32	92.88	92.88	92.1	96.77	89.10	96.44	95.55	88.77	94.33
	Ch 4	No	1.11	4.56	6.12	3.56	2.56	3.67	3.34	1.45	0.89	3.78	3.23	5.67	4.23	2.45	4.56
		Yes	93.66	75.31	85.54	86.87	81.31	76.31	82.76	69.19	84.76	91.43	76.97	91.21	77.75	87.88	84.43
	Ch 5	No	1.89	4.12	3.11	4.67	0.78	3.45	3.45	3.56	1.00	3.45	2.89	2.34	3.45	3.11	1.78
		Yes	97.55	89.43	89.21	80.65	86.43	77.98	88.88	83.76	85.54	90.77	78.53	83.87	92.77	85.87	87.54
	Ch 6	No	4.23	2.34	3.00	3.23	3.23	2.56	3.56	1.33	3.23	4.12	1.78	3.23	5.12	4.34	1.11
		Yes	94.33	90.77	86.43	80.42	80.20	77.09	86.21	82.31	84.76	80.76	83.43	78.31	89.32	80.65	84.09
	Ch 7	No	3.23	2.34	3.34	4.45	3.45	3.23	3.11	3.34	6.12	2.22	6.12	2.34	3.45	3.11	0.89
		Yes	91.88	87.88	90.10	82.76	71.97	85.87	81.09	43.38	83.54	80.98	84.32	81.31	84.43	76.20	90.32
	Ch 8	No	3.45	5.34	3.78	3.78	3.23	3.56	1.00	6.90	3.11	5.56	2.11	5.90	0.44	2.45	4.12
		Yes	85.76	83.98	88.21	86.21	83.76	87.76	82.54	35.93	84.98	78.42	74.08	74.08	82.42	79.98	85.43
	Ch 9	No	3.89	1.89	2.00	1.89	2.11	3.34	3.00	4.78	1.22	3.45	4.78	1.22	0.56	4.56	0.67
		Yes	72.08	72.41	81.76	72.64	69.52	73.75	84.09	75.75	63.85	84.32	65.29	78.64	83.43	66.63	82.87
	Ch 10	No	1.89	2.56	3.56	3.56	2.56	3.56	2.34	2.22	3.67	3.45	5.01	3.11	2.34	1.78	1.67
		Yes	82.98	73.97	78.20	82.76	79.87	66.74	75.53	71.52	83.09	85.98	53.5	64.96	82.87	64.52	69.97
	Ch 11	No	9.12	3.23	5.45	3.11	3.34	2.45	3.67	2.67	4.89	4.00	3.23	1.67	6.12	5.01	2.56
		Yes	88.77	78.87	77.31	76.75	78.31	70.75	83.76	70.3	84.09	76.64	69.08	92.55	76.86	73.97	81.87
	Ch 12	No	2.67	5.01	5.12	3.67	2.89	3.45	1.00	2.78	4.45	5.67	3.45	5.23	1.56	3.23	3.34
		Yes	82.87	80.20	77.64	62.29	68.85	72.97	85.43	80.53	71.75	83.54	65.07	80.65	72.97	69.52	76.97
	Ch 13	No	1.11	5.34	2.00	2.22	4.89	2.56	5.78	3.78	1.67	3.34	3.45	0.44	2.56	1.56	6.01
		Yes	92.55	69.74	63.29	78.75	58.06	51.61	85.98	79.64	66.96	74.30	76.08	76.2	50.83	65.63	87.99
	Ch 14	No	5.34	5.90	2.00	8.79	1.67	2.45	3.67	2.67	3.56	5.78	5.34	6.01	3.11	1.11	1.67
		Yes	81.09	61.74	67.41	74.86	75.42	42.94	86.21	63.96	72.19	84.32	71.30	74.64	85.87	71.30	85.21
	Ch 15	No	3.23	0.56	4.67	3.67	3.23	3.34	2.67	8.68	2.56	3.89	3.23	4.67	5.67	2.78	4.56
		Yes	86.10	71.86	69.86	72.75	75.86	20.24	77.98	73.86	70.97	78.64	66.30	66.07	73.30	75.86	65.96
	Ch 16	No	3.89	3.56	1.00	3.34	8.01	3.67	2.45	3.34	3.11	3.23	3.34	1.67	3.89	3.34	3.34
		Yes	81.54	55.06	85.21	70.41	74.97	74.86	77.75	79.76	72.53	75.97	70.75	65.85	67.19	83.43	71.08
	**Mean**	**No**	**3.73**	**3.19**	**3.03**	**4.32**	**3.03**	**3.30**	**3.19**	**3.66**	**3.18**	**3.83**	**4.03**	**3.44**	**3.55**	**3.12**	**2.90**
		**Yes**	**88.38**	**79.77**	**82.91**	**80.40**	**76.81**	**72.62**	**85.41**	**74.00**	**81.02**	**84.42**	**75.44**	**80.12**	**81.53**	**78.07**	**83.60**
	**SD**	**No**	**2.04**	**1.71**	**1.68**	**2.03**	**1.66**	**0.53**	**1.20**	**2.02**	**1.54**	**1.02**	**1.73**	**1.86**	**2.01**	**1.14**	**1.82**
		**Yes**	**7.02**	**12.13**	**10.03**	**8.83**	**7.26**	**20.01**	**6.45**	**15.77**	**10.29**	**7.12**	**10.71**	**9.93**	**11.78**	**8.91**	**8.82**
4 chs	Ch 1-4	No	1.56	4.67	3.89	2.89	0.56	2.56	2.89	3.34	3.45	5.78	0.78	3.78	0.89	2.22	2.56
		Yes	96.89	99.11	98.22	93.99	86.10	97.44	96.11	93.88	95.22	99.11	90.55	99.44	95.77	94.66	95.55
	Ch 5-8	No	3.23	3.23	3.23	3.23	3.23	3.23	3.23	2.45	3.23	3.23	3.23	3.23	3.23	4.34	3.23
		Yes	97.00	92.21	90.55	86.43	88.32	88.88	87.21	84.43	85.09	85.32	84.32	83.54	88.43	85.32	89.21
	Ch 9-12	No	3.00	3.45	1.22	2.34	2.22	3.00	1.33	4.89	2.00	1.11	3.45	3.34	3.89	3.45	3.78
		Yes	84.20	82.98	84.76	83.2	80.42	76.31	90.21	83.31	76.86	90.21	71.86	87.54	85.32	73.19	89.77
	Ch 13-16	No	4.12	3.56	5.01	2.78	5.78	3.11	4.12	2.22	3.45	3.67	2.11	3.34	2.78	2.45	3.78
		Yes	92.77	77.98	75.75	85.43	80.76	70.41	83.76	81.54	76.53	88.54	79.42	77.31	70.52	83.43	78.42
	**Mean**	**No**	**2.98**	**3.73**	**3.34**	**2.81**	**2.95**	**2.98**	**2.89**	**3.23**	**3.03**	**3.45**	**2.39**	**3.42**	**2.70**	**3.12**	**3.34**
		**Yes**	**92.72**	**88.07**	**87.32**	**87.26**	**83.90**	**83.26**	**89.32**	**85.79**	**83.43**	**90.80**	**81.54**	**86.96**	**85.01**	**84.15**	**88.24**
	**SD**	**No**	**1.06**	**0.64**	**1.59**	**0.37**	**2.19**	**0.29**	**1.16**	**1.21**	**0.70**	**1.91**	**1.22**	**0.24**	**1.29**	**0.98**	**0.58**
		**Yes**	**6.01**	**9.43**	**9.48**	**4.68**	**3.93**	**12.19**	**5.24**	**5.52**	**8.80**	**5.90**	**7.90**	**9.33**	**10.61**	**8.80**	**7.14**
**4 sites**	**Ch 1-16**	**No**	**1.67**	**2.22**	**3.34**	**6.01**	**6.45**	**3.34**	**1.67**	**4.23**	**5.45**	**1.33**	**3.56**	**4.12**	**2.89**	**1.00**	**5.56**
		**Yes**	**98.22**	**95.44**	**97.55**	**96.89**	**93.66**	**91.21**	**99.22**	**97.55**	**94.44**	**97.00**	**92.10**	**94.10**	**92.55**	**91.21**	**93.99**

Table S9. Accuracy (%) of cross-session experiments and new user experiments with and without calibration.

Gestures	Intended commands	Attempt No.	End-to-end delay (ms)	Recognized commands	Correct rate
Rest	No Demand	1	275	No Demand	100.0%
		2	58	No Demand	
		3	225	No Demand	
		4	333	No Demand	
		5	267	No Demand	
		6	167	No Demand	
		7	433	No Demand	
		8	300	No Demand	
Index finger 1cm	Mild Pain	1	100	Mild Pain	75.0%
		2	200	Mild Pain	
		3	67	Mild Pain	
		4	367	No Demand	
		5	500	Mild Pain	
		6	275	Mild Pain	
		7	67	No Demand	
		8	175	Mild Pain	
Index finger 2cm	Moderate Pain	1	100	Moderate Pain	75.0%
		2	175	Moderate Pain	
		3	67	Moderate Pain	
		4	367	Severe Pain	
		5	200	Moderate Pain	
		6	467	Moderate Pain	
		7	433	Moderate Pain	
		8	100	Mild Pain	
Index finger 3cm	Severe Pain	1	300	Severe Pain	87.5%
		2	100	Severe Pain	
		3	600	Moderate Pain	
		4	200	Severe Pain	
		5	433	Severe Pain	
		6	200	Severe Pain	
		7	275	Severe Pain	
		8	333	Severe Pain	
Thumb 1cm	Mild Respiratory Distress	1	33	Severe Respiratory Distress	87.5%
		2	100	Mild Respiratory Distress	
		3	167	Mild Respiratory Distress	
		4	300	Mild Respiratory Distress	
		5	233	Mild Respiratory Distress	
		6	175	Mild Respiratory Distress	
		7	533	Mild Respiratory Distress	
		8	233	Mild Respiratory Distress	
Thumb 3cm	Severe Respiratory Distress	1	333	Severe Respiratory Distress	75.0%
		2	175	Severe Pain	
		3	33	Severe Respiratory Distress	
		4	200	Severe Respiratory Distress	
		5	33	Severe Respiratory Distress	
		6	367	Severe Pain	
		7	100	Severe Respiratory Distress	
		8	433	Severe Respiratory Distress	
Ring finger 2cm	Emergency Call	1	100	Emergency Call	100.0%
		2	100	Emergency Call	
		3	100	Emergency Call	
		4	67	Emergency Call	
		5	200	Emergency Call	
		6	267	Emergency Call	
		7	233	Emergency Call	
		8	67	Emergency Call	

Table S10. Command-level performance of the assistive non-verbal communication interface.

No.	Piezoelectric Material Type	Backing Layer (Yes/No)	Matching Layer (Yes/No)	Flexible (Yes/No)	Center Frequency (MHz)	Bandwidth Definition	Relative Bandwidth (%)	Absolute Bandwidth (MHz)	Referen-ce
1	1-3 composite	NO	NO	YES	7.5	-6dB	32%	2.40	*(12)*
2	1-3 composite	YES	NO	YES	3.51	-6dB	47.11%	1.65	*(45)*
3	1-3 composite				2.8	-6dB	~50%	1.40	*(46)*
4	1-3 composite				3	-6dB	55%	1.65	*(28)*
5	1-3 composite				~4	-6dB	40-45%	1.60-1.80	*(37)*
6	1-3 composite				5	-6dB	**49.23%**	**2.46**	**This work**
7	2-2 composite	YES	YES	YES	7.5	-6dB	65%	4.88	*(16)*
8	1-3 composite				~5	-6dB	46.8%	2.34	*(47)*
9	1-3 composite	YES	YES	NO	10	-6dB	90.3%	9.03	*(21)*
10	1-3 composite				3/7/10	-6dB	68/75/78%	2.04/5.25/7.80	*(27)*
11	PMN-PT				7.1	-6dB	70%	4.97	*(48)*

Table S11. Comparison of bandwidth of different wearable ultrasound devices.

Movie S1. Non-verbal communication human-machine interface based on fine-grained multi-level gesture recognition.

REFERENCES

1. P. Tan, X. Han, Y. Zou, X. Qu, J. Xue, T. Li, Y. Wang, R. Luo, X. Cui, Y. Xi, L. Wu, B. Xue, D. Luo, Y. Fan, X. Chen, Z. Li, Z. L. Wang, Self-powered gesture recognition wristband enabled by machine learning for full keyboard and multicommand input. *Adv. Mater.* **34**, 2200793 (2022).

2. F. Wen, Z. Sun, T. He, Q. Shi, M. Zhu, Z. Zhang, L. Li, T. Zhang, C. Lee, Machine learning glove using self-powered conductive superhydrophobic triboelectric textile for gesture recognition in VR/AR applications. *Adv. Sci.* **7**, 2000261 (2020).

3. H. Zhou, W. Huang, Z. Xiao, S. Zhang, W. Li, J. Hu, T. Feng, J. Wu, P. Zhu, Y. Mao, Deep-learning-assisted noncontact gesture-recognition system for touchless human-machine interfaces. *Adv. Funct. Mater.* **32**, 2208271 (2022).

4. Q. Huang, Y. Jiang, Z. Duan, Z. Yuan, Y. Wu, J. Peng, Y. Xu, H. Li, H. He, H. Tai, A finger motion monitoring glove for hand rehabilitation training and assessment based on gesture recognition. *IEEE Sens. J.* **23**, 13789–13796 (2023).

5. W. Li, S. Wu, S. Li, X. Zhong, X. Zhang, H. Qiao, M. Kang, J. Chen, P. Wang, L.-Q. Tao, Gesture recognition system using reduced graphene oxide-enhanced hydrogel strain sensors for rehabilitation training. *ACS Appl. Mater. Interfaces* **15**, 45106–45115 (2023).

6. H. P, A. V, A. K, T. R. H. Subramaniam, J. J. Nair, Vision based gesture recognition. *Procedia Comput. Sci* **235**, 303–315 (2024).

7. H. P. Wang, Y. X. Zhou, H. Li, G. D. Liu, S. M. Yin, P. J. Li, S. Y. Dong, C. Y. Gong, S. Y. Wang, Y. B. Li, T. J. Cui, Noncontact electromagnetic wireless recognition for prosthesis based on intelligent metasurface. *Adv. Sci.* **9**, e2105056 (2022).

8. Q. Ma, Z. Gu, X. Gao, L. Chen, S. L. Qin, Q. W. Wu, Q. Xiao, J. W. You, T. J. Cui, Intelligent hand-gesture recognition based on programmable topological metasurfaces. *Adv. Funct. Mater.* **35**, 2411667 (2025).

9. D. Zhang, Z. Liao, W. Xie, X. Wu, H. Xie, J. Xiao, L. Jiang, Fine-grained and real-time gesture recognition by using IMU sensors. *IEEE Trans. Mob. Comput.* **22**, 2177–2189 (2023).

10. S. Wang, T. Zhang, Y. Li, P. Li, H. Wu, K. Li, Continuous hand gestures detection and recognition in emergency human-robot interaction based on the inertial measurement unit. *IEEE Trans. Instrum. Meas.* **73**, 1–15 (2024).

11. A. Moin, A. Zhou, A. Rahimi, A. Menon, S. Benatti, G. Alexandrov, S. Tamakloe, J. Ting, N. Yamamoto, Y. Khan, F. Burghardt, L. Benini, A. C. Arias, J. M. Rabaey, A wearable biosensing system with in-sensor adaptive machine learning for hand gesture recognition. *Nat. Electron.* **4**, 54–63 (2021).

12. C. Wang, W. Hong, Y. Deng, L. Lan, S. Zhang, J. Ping, Y. Ying, C. Yu, J. Luo, W. Chen, Z. Chen, J. Song, Wireless, wrist-worn ultraconformal neuromuscular interfaces for thumb-controlled immersive human–machine interactions. *Adv. Funct. Mater.* **35**, 2422980 (2025).

13. H. Lee, S. Lee, J. Kim, H. Jung, K. J. Yoon, S. Gandla, H. Park, S. Kim, Stretchable array electromyography sensor with graph neural network for static and dynamic gestures recognition system. *Npj Flex. Electron.* **7**, 20 (2023).

14. P. Kaifosh, T. R. Reardon, CTRL-labs at Reality Labs, A generic non-invasive neuromotor interface for human-computer interaction. *Nature* **645**, 702–711 (2025).

15. Y. Zhao, C. Chen, B. Lu, X. Zhu, G. Gu, All 3D-printed soft high-density surface electromyography electrode arrays for accurate muscle activation mapping and decomposition. *Adv. Funct. Mater.* **34**, 2312480 (2024).

16. H. Zhao, W. Chen, Y. Li, H. Wang, H. Li, T. Li, F. Han, J. Sun, L. Huang, X. Peng, J. Chen, Y. Yang, X. Qiu, Y. Liu, H. Yu, W. Hou, Q. Li, G. Fu, C. You, X. Liu, F. Li, X. Li, G. Zhao, L. Wang, P. Fang, G. Li, H. Zheng, M. Zhu, W. Yan, Q. Tian, T. Ma, Z. Liu, In situ structural-functional synchronous dissection of dynamic neuromuscular system via an integrated multimodal wearable patch. *Sci. Adv.* **11**, eads1486 (2025).

17. S. Zhou, G. Park, M. Lin, X. Yang, S. Xu, Wearable ultrasound technology. *Nat. Rev. Bioeng.* **3**, 835–854 (2025).

18. J. Shi, J.-Y. Guo, S.-X. Hu, Y.-P. Zheng, Recognition of finger flexion motion from ultrasound image: A feasibility study. *Ultrasound Med. Biol.* **38**, 1695–1704 (2012).

19. S. Sikdar, H. Rangwala, E. B. Eastlake, I. A. Hunt, A. J. Nelson, J. Devanathan, A. Shin, J. J. Pancrazio, Novel method for predicting dexterous individual finger movements by imaging muscle activity using a wearable ultrasonic system. *IEEE Trans. Neural Syst. Rehabil. Eng.* **22**, 69–76 (2014).

20. N. Akhlaghi, C. A. Baker, M. Lahlou, H. Zafar, K. G. Murthy, H. S. Rangwala, J. Kosecka, W. M. Joiner, J. J. Pancrazio, S. Sikdar, Real-time classification of hand motions using ultrasound imaging of forearm muscles. *I.E.E.E. Trans. Biomed. Eng.* **63**, 1687–1698 (2016).

21. G. Lu, S. Kim, X. Chen, Y. Zeng, D. Li, S. Wang, B. Liu, S. Li, R. Li, B. Deng, J. Zhang, C. Gong, A. P. Chandrakasan, Q. Zhou, X. Zhao, Hand tracking using wearable wrist imaging. *Nat. Electron.* **9**, 426–440 (2026).

22. Y. Huang, X. Yang, Y. Li, D. Zhou, K. He, H. Liu, Ultrasound-based sensing models for finger motion classification. *IEEE J. Biomed. Health Inform.* **22**, 1395–1405 (2018).

23. W. Xia, Y. Zhou, X. Yang, K. He, H. Liu, Toward portable hybrid surface electromyography/A-mode ultrasound sensing for human–machine interface. *IEEE Sens. J.* **19**, 5219–5228 (2019).

24. J. Zeng, Y. Zhou, Y. Yang, J. Yan, H. Liu, Fatigue-sensitivity comparison of sEMG and A-mode ultrasound based hand gesture recognition. *IEEE J. Biomed. Health Inform.* **26**, 1718–1725 (2022).

25. X. Yang, Y. Zhou, H. Liu, Wearable ultrasound-based decoding of simultaneous wrist/hand kinematics. *IEEE Trans. Ind. Electron.* **68**, 8667–8675 (2021).

26. X. Yang, Y. Liu, Z. Yin, P. Wang, P. Deng, Z. Zhao, H. Liu, Simultaneous prediction of wrist and hand motions via wearable ultrasound sensing for natural control of hand prostheses. *IEEE Trans. Neural Syst. Rehabil. Eng.* **30**, 2517–2527 (2022).

27. C. Wang, X. Chen, L. Wang, M. Makihata, H.-C. Liu, T. Zhou, X. Zhao, Bioadhesive ultrasound for long-term continuous imaging of diverse organs. *Science* **377**, 517–523 (2022).

28. H. Hu, H. Huang, M. Li, X. Gao, L. Yin, R. Qi, R. S. Wu, X. Chen, Y. Ma, K. Shi, C. Li, T. M. Maus, B. Huang, C. Lu, M. Lin, S. Zhou, Z. Lou, Y. Gu, Y. Chen, Y. Lei, X. Wang, R. Wang, W. Yue, X. Yang, Y. Bian, J. Mu, G. Park, S. Xiang, S. Cai, P. W. Corey, J. Wang, S. Xu, A wearable cardiac ultrasound imager. *Nature* **613**, 667–675 (2023).

29. M. Lin, Z. Zhang, X. Gao, Y. Bian, R. S. Wu, G. Park, Z. Lou, Z. Zhang, X. Xu, X. Chen, A. Kang, X. Yang, W. Yue, L. Yin, C. Wang, B. Qi, S. Zhou, H. Hu, H. Huang, M. Li, Y. Gu, J. Mu, A. Yang, A. Yaghi, Y. Chen, Y. Lei, C. Lu, R. Wang, J. Wang, S. Xiang, E. B. Kistler, N. Vasconcelos, S. Xu, A fully integrated wearable ultrasound system to monitor deep tissues in moving subjects. *Nat. Biotechnol.* **42**, 448–457 (2024).

30. C.-C. Yu, A. Shah, N. Amiri, C. Marcus, M. O. G. Nayeem, A. K. Bhayadia, A. Karami, C. Dagdeviren, A conformable ultrasound patch for cavitation-enhanced transdermal cosmeceutical delivery. *Adv. Mater.* **35**, e2300066 (2023).

31. H. Liu, S. Ding, X. Lin, S. Wang, Y. Wang, Z. Feng, J. Song, Bone fracture healing under the intervention of a stretchable ultrasound array. *ACS Nano* **18**, 19549–19560 (2024).

32. H. Liu, J. Fan, X. Lin, K. Lin, S. Wang, S. Liu, F. Wang, J. Song, Identification of gas-liquid two-phase flow patterns based on flexible ultrasound array and machine learning. *Npj Flex. Electron.* **8**, 67 (2024).

33. C. Wang, X. Li, H. Hu, L. Zhang, Z. Huang, M. Lin, Z. Zhang, Z. Yin, B. Huang, H. Gong, S. Bhaskaran, Y. Gu, M. Makihata, Y. Guo, Y. Lei, Y. Chen, C. Wang, Y. Li, T. Zhang, Z. Chen, A. P. Pisano, L. Zhang, Q. Zhou, S. Xu, Monitoring of the central blood pressure waveform via a conformal ultrasonic device. *Nat. Biomed. Eng* **2**, 687–695 (2018).

34. F. Wang, P. Jin, Y. Feng, J. Fu, P. Wang, X. Liu, Y. Zhang, Y. Ma, Y. Yang, A. Yang, X. Feng, Flexible Doppler ultrasound device for the monitoring of blood flow velocity. *Sci. Adv.* **7**, eabi9283 (2021).

35. Q. Wang, Y. Zhang, H. Xue, Y. Zeng, G. Lu, H. Fan, L. Jiang, J. Wu, Lead-free dual-frequency ultrasound implants for wireless, biphasic deep brain stimulation. *Nat. Commun.* **15**, 4017 (2024).

36. P. Jin, J. Fu, F. Wang, Y. Zhang, P. Wang, X. Liu, Y. Jiao, H. Li, Y. Chen, Y. Ma, X. Feng, A flexible, stretchable system for simultaneous acoustic energy transfer and communication. *Sci. Adv.* **7**, eabg2507 (2021).

37. X. Gao, X. Chen, M. Lin, W. Yue, H. Hu, S. Qin, F. Zhang, Z. Lou, L. Yin, H. Huang, S. Zhou, Y. Bian, X. Yang, Y. Zhu, J. Mu, X. Wang, G. Park, C. Lu, R. Wang, R. S. Wu, J. Wang, J. Li, S. Xu, A wearable echomyography system based on a single transducer. *Nat. Electron.* **7**, 1035–1046 (2024).

38. M. Cai, H. Yang, L. Shen, S. Nie, Z. Mao, C. Gao, Y. Zhu, J. Song, Implantable thermal therapeutic device with precise temperature control enabled by foldable electronics and heat-insulating pads. *Research* **2022**, 9787296 (2022).

39. X. Yang, X. Sun, D. Zhou, Y. Li, H. Liu, Towards wearable A-mode ultrasound sensing for real-time finger motion recognition. *IEEE Trans. Neural Syst. Rehabil. Eng.* **26**, 1199–1208 (2018).

40. X. Yang, Z. Chen, N. Hettiarachchi, J. Yan, H. Liu, A wearable ultrasound system for sensing muscular morphological deformations. *IEEE Trans. Syst. Man Cybern. Syst.* **51**, 3370–3379 (2021).

41. J. Zeng, Y. Sheng, Z. Zhou, Y. Yang, J. Wang, Q. Xie, H. Liu, Adaptive learning against muscle fatigue for A-mode ultrasound-based gesture recognition. *IEEE Trans. Instrum. Meas.* **72**, 1–12 (2023).

42. X. Yang, J. Yan, Z. Chen, H. Ding, H. Liu, A proportional pattern recognition control scheme for wearable A-mode ultrasound sensing. *IEEE Trans. Ind. Electron.* **67**, 800–808 (2020).

43. Y. Jin, J. T. Alvarez, E. L. Suitor, K. Swaminathan, A. Chin, U. S. Civici, R. W. Nuckols, R. D. Howe, C. J. Walsh, Estimation of joint torque in dynamic activities using wearable A-mode ultrasound. *Nat. Commun.* **15**, 5756 (2024).

44. Z. Yin, H. Chen, X. Yang, Y. Liu, N. Zhang, J. Meng, H. Liu, A wearable ultrasound interface for prosthetic hand control. *IEEE J. Biomed. Health Inform.* **26**, 5384–5393 (2022).

45. H. Hu, X. Zhu, C. Wang, L. Zhang, X. Li, S. Lee, Z. Huang, R. Chen, Z. Chen, C. Wang, Y. Gu, Y. Chen, Y. Lei, T. Zhang, N. Kim, Y. Guo, Y. Teng, W. Zhou, Y. Li, A. Nomoto, S. Sternini, Q. Zhou, M. Pharr, F. L. di Scalea, S. Xu, Stretchable ultrasonic transducer arrays for three-dimensional imaging on complex surfaces. *Sci. Adv.* **4**, eaar3979 (2018).

46. H. Hu, Y. Ma, X. Gao, D. Song, M. Li, H. Huang, X. Qian, R. Wu, K. Shi, H. Ding, M. Lin, X. Chen, W. Zhao, B. Qi, S. Zhou, R. Chen, Y. Gu, Y. Chen, Y. Lei, C. Wang, C. Wang, Y. Tong, H. Cui, A. Abdal, Y. Zhu, X. Tian, Z. Chen, C. Lu, X. Yang, J. Mu, Z. Lou, M. Eghtedari, Q. Zhou, A. Oberai, S. Xu, Stretchable ultrasonic arrays for the three-dimensional mapping of the modulus of deep tissue. *Nat. Biomed. Eng* **7**, 1321–1334 (2023).

47. S. Yin, H. Zhang, F. Shi, Y. Chen, C. Zhong, R. Li, Y. Su, Bendable phased-array ultrasound transducer for imaging on curved surfaces. *ACS Nano* **19**, 8030–8039 (2025).

48. W. Du, L. Zhang, E. Suh, D. Lin, C. Marcus, L. Ozkan, A. Ahuja, S. Fernandez, I. I. Shuvo, D. Sadat, W. Liu, F. Li, A. P. Chandrakasan, T. Ozmen, C. Dagdeviren, Conformable ultrasound breast patch for deep tissue scanning and imaging. *Sci. Adv.* **9**, eadh5325 (2023).

49. A. H. Al-Timemy, G. Bugmann, J. Escudero, N. Outram, Classification of finger movements for the dexterous hand prosthesis control with surface electromyography. *IEEE J. Biomed. Health Inform.* **17**, 608–618 (2013).

50. J. Liu, P. Zhou, A novel myoelectric pattern recognition strategy for hand function restoration after incomplete cervical spinal cord injury. *IEEE Trans. Neural Syst. Rehabil. Eng.* **21**, 96–103 (2013).

51. N. Tacca, C. Dunlap, S. P. Donegan, J. O. Hardin, E. Meyers, M. J. Darrow, S. Colachis Iv, A. Gillman, D. A. Friedenberg, Wearable high-density EMG sleeve for complex hand gesture classification and continuous joint angle estimation. *Sci. Rep.* **14**, 18564 (2024).

52. P. Sri-iesaranusorn, A. Chaiyaroj, C. Buekban, S. Dumnin, R. Pongthornseri, C. Thanawattano, D. Surangsrirat, Classification of 41 hand and wrist movements via surface electromyogram using deep neural network. *Front. Bioeng. Biotechnol.* **9**, 548357 (2021).

53. K. K. Kim, T. J. Zaluska, S. Skov, Y. Lee, H. Park, D. Zhong, M. Khatib, Y. Nishio, Y. Jiang, S. L. Delp, Z. Bao, A simplified wearable device powered by a generative EMG network for hand-gesture recognition and gait prediction. *Nat. Sens.* **1**, 27–38 (2026).

54. X. Yang, D. Zhou, Y. Zhou, Y. Huang, H. Liu, Towards zero re-training for long-term hand gesture recognition via ultrasound sensing. *IEEE J. Biomed. Health Inform.* **23**, 1639–1646 (2019).

55. M. Qu, D. Lv, J. Zhou, Z. Wang, Y. Zheng, G. Zhang, J. Xie, Sensing and controlling strategy for upper extremity prosthetics based on piezoelectric micromachined ultrasound transducer. *IEEE Trans. Biomed. Eng.* **71**, 1161–1169 (2023).

56. L. Guo, Z. Lu, L. Yao, S. Cai, A gesture recognition strategy based on A-mode ultrasound for identifying known and unknown gestures. *IEEE Sens. J.* **22**, 10730–10739 (2022).

57. Z. Tang, Y. Wu, M. Qu, J. Xu, W. Liu, Y. Zheng, J. Xie, Synchronous gesture recognition and arm joint angle monitoring for human-machine interaction using multiple flexible ultrasonic patches. *Adv. Funct. Mater.* **35**, e05131 (2025).

58. J. He, H. Luo, J. Jia, J. T. W. Yeow, N. Jiang, Wrist and finger gesture recognition with single-element ultrasound signals: A comparison with single-channel surface electromyogram. *IEEE Trans. Biomed. Eng.* **66**, 1277–1284 (2019).
